# Nanomaterial-mediated strategies for enhancing bioremediation of polycyclic aromatic hydrocarbons: A systematic review

**DOI:** 10.1016/j.hybadv.2024.100315

**Published:** 2024-12

**Authors:** Nitu Gupta, Apurba Koley, Sandipan Banerjee, Anudeb Ghosh, Raza Rafiqul Hoque, Srinivasan Balachandran

**Affiliations:** a Department of Environmental Science, Tezpur University, Tezpur 784028, Assam, India; b Department of Environmental Studies, Visva-Bharati, Santiniketan 731235, West Bengal, India; c Faculty of Forestry and Wood Sciences, Czech University of Life Sciences Prague, Kamýcká 129, Prague 165 00, Czech Republic

**Keywords:** *PAHs*, *Bioremediation*, *Nano-bioremediation*, *Nanoparticle enhanced microbial activity*, *Biogenic nanomaterial-Mediated remediation*

## Abstract

Polycyclic aromatic hydrocarbons (PAHs) are pervasive organic pollutants in the environment that are formed as an outcome of partial combustion of organic matter. PAHs pose a significant threat to ecological systems and human health due to their cytotoxic and genotoxic effects. Therefore, an immediate need for effective PAH remediation methods is crucial. Although nanomaterials are effective for remediation of PAHs, concerns regarding environmental compatibility and sustainability remains. Therefore, this study emphasizes integration of nanomaterials with bioremediation methods, which might offer a more sustainable and ecofriendly approach to PAHs remediation. A systematic search was conducted through scholarly databases from 2013 to 2023. A total of 360 articles were scrutinized, among which 26 articles were selected that resonated with the application of nano-bioremediation. These literatures comprise both comparative analysis of bioremediation only as well as nano-bioremediation. There is an elevation of 18.9 % in PAHs removal of liquid-phase samples, when comparing bioremediation (52.2 %) with nano-bioremediation (71.1 %). A consistent trend was observed in soil samples, with bioremediation and nano-bioremediation that successfully remove PAHs, with 60.8 % and 75.1 % respectively, indicating a 14.3 % improvement. Furthermore, the review elaborated on the various features of nanomaterials that led to their efficiency in the bioremediation of PAH. The review also discussed the strategies of nano-bioremediation namely nanomaterial-assisted microbial degradation, nanomaterial-assisted enzyme-enhanced microbial activity, nanomaterial-immobilized microbial cells, nanomaterial-facilitated electron transfer, and even some eco-green approaches to remediate PAHs, like biogenic nanomaterial for PAHs.

## 1 Introduction

The environment is getting increasingly contaminated with xenobiotic pollutants, including polycyclic aromatic hydrocarbons (PAHs) mainly due to the expansion and rapid rise in industrialization. These hazardous PAHs are ubiquitous and pose a potential threat to ecosystems including human beings as they are mutagenic, carcinogenic, and endocrine disruptors [1]. Sixteen PAHs are recorded as priority contaminants by the United States Environmental Protection Agency (USEPA) because of their deleterious effects on ecosystems [2]. PAHs are a group of organic compounds composed of two/more merged benzenoid rings. They are ubiquitous in the environment that originate both from natural phenomena such as fires and volcanic activities, and from anthropogenic sources like fossil fuel combustion, carbon black processing, coal tar, and petroleum seepage [[3], [4], [5], [6], [7]]. More aromatic rings, more structural angularity, and higher hydrophobicity make PAHs more electrochemically stable, persistent, and biodegradation resistant making them difficult to degrade [2,8].

Zhang and Tao [9] stated that the global PAHs emission was 530,000 tons into the atmosphere resulting from various man-made actions like biofuel combustion (56.7 %) and wildfire (17 %) in 2004. While traffic fuel, and domestic coal burning accounted for 4.8 %, and 3.7 % respectively. China is the major contributor of PAHs emission with 114,000 tons, followed by India with 90,000 tons, the USA contributing 32,000 tons [9,10]. Through dry or wet deposition processes, atmospheric PAHs accumulate in water, soil, sediment, and vegetation [11,12]. In fact, soil and sediment are considered as long-term repository of PAHs [13,14] and, as such, soil can be considered as a representative of the pollution status with respect to the PAHs [15]. The low vapor pressure and high hydrophobicity of PAHs higher benzenoid rings result in their intense adsorption to soil particles [16]. In research by Hoffman et al. [17] 79 %–93 % of PAHs were linked with suspended solids, indicating that their physical state is mostly solid. The absorption and translocation of PAHs by plants can result in groundwater and food contamination as a result of their accumulation in soil. Exposure to PAHs is inevitable and primarily occurs through ingestion, inhalation, and dermal contact [2]. One cigarette can introduce 20–40 ng of benzo [a]pyrene to smokers, while non-smokers can expose themselves to up to 70 % of their PAHs through their diet [18].

**Fig. 1:**
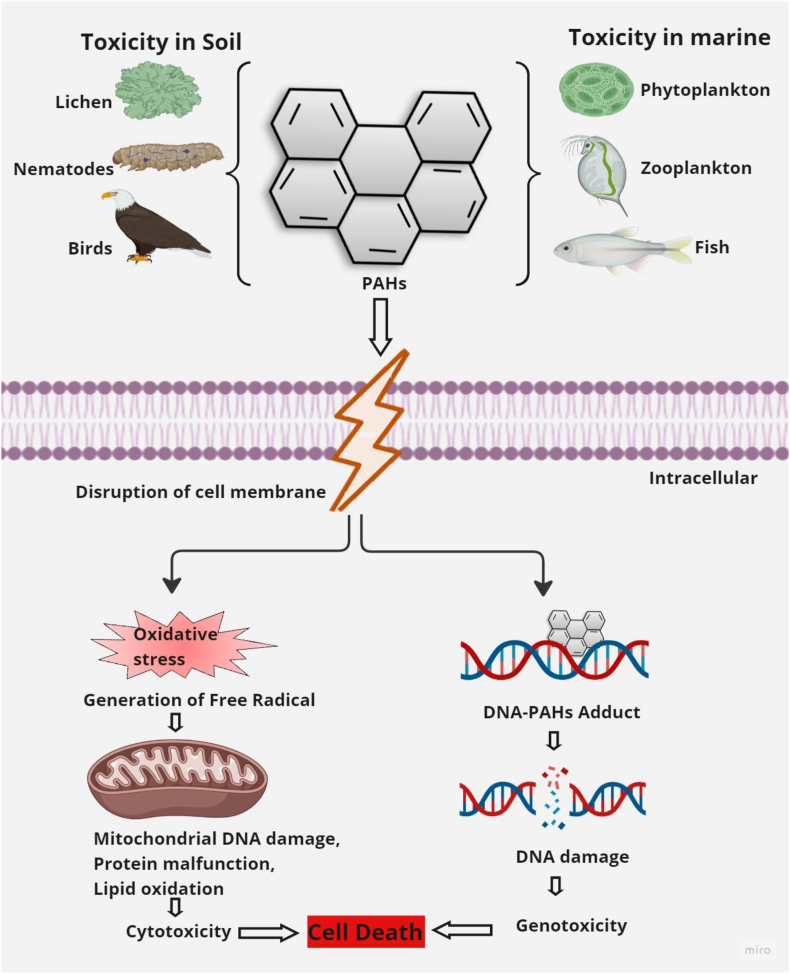
Toxicity of PAHs in soil and marine ecosystem.

Conventionally, PAHs are categorized into two groups based on their molecular weight. The low molecular weight PAHs (LMW-PAHs) include naphthalene (NAP), acenaphthene (ACE), fluorene (FLR), phenanthrene (PHE), and anthracene (ANT), all of which contain fewer than four rings. The high molecular weight PAHs (HMW-PAHs), which contain four or more rings include compounds such as pyrene (PYR), chrysene (CHRY), fluoranthene (FLU), benzo [a]anthracene (BAA), benzo [a]pyrene (BAP), benzo [b]fluoranthene (BBF), benzo [k]fluoranthene (BKF), dibenzo [a,h]anthracene (DBA), benzo [g,h,i]perylene (BghiP), and indeno [1,2,3-c,d]pyrene (ICP) [19]. HMW-PAHs, including PYR, BAP, and BBF, are generally resistant to microbial degradation due to their low solubility and bioavailability, making them persistent in the environment, resistant to degradation and prone to bioaccumulation [20]. BAP is linked to cardiopulmonary disorders, psychiatric conditions, and various cancers, including skin, lung, bladder, and gastrointestinal cancers [21,22]. NAP, PYR, FLR, and ANT have been associated with pulmonary disorders, while CHRY induces oxidative stress and cytotoxicity in human hepatocytes [23]. BAA, BAP, and NAP are known to be embryotoxic as reported in animal studies [21]. Skin contact with PAHs like NAP, ANT, and BAP can cause irritation, allergic reactions, and inflammation [22]. PAHs such as PHE and FLR induce oxidative stress and inflammation in human lung epithelial cells [24]. PAHs are associated with skin, bladder, lung, and gastrointestinal cancers. Short term exposure to PAHs leads to headaches, nausea, and eye irritation, whereas long-term contact causes respiratory difficulties, lung infection, asthma, and various cancers [25,26]. PAHs are carcinogenic due to the presence of bay or K region in their molecular structure, directly associated with the generation of bay or K region epoxides, which are extremely reactive and have high affinity towards mammalian DNA [27]. Furthermore, this might lead to the creation of DNA adducts, which can alter normal cells into tumorigenic cells, exacerbating the health hazards linked to PAHs [16]. Fig. 1 displays the interactions between PAHs and DNA and other cellular organelles, specifically demonstrating cytotoxicity and genotoxicity. Remediation measures are implemented to address these problems, combat PAH contamination, and protect both humans and the environment [28]. Several techniques for reducing the toxicity of PAHs by degradation or transformation, including physical, chemical, and biological mechanisms that can be used to protect the ecosystem and eradicate the toxicity of PAH contamination, which is crucial for remediation [29].

Nanomaterials have drawn significant attention due to their unique properties which motivates for potential applications, particularly in bioremediation [30]. Their small size and large surface area-to-volume ratio offer distinct advantages for increasing the adsorption and biodegradation of hazardous contaminants like PAHs in contaminated soil [31,32]. However, the nanomaterials have certain limitation such as nanotoxicity, antimicrobial properties, high production cost, and poor eco-sustainability. Therefore, nanomaterials such as carbon nanotubes (CNTs), biopolymers, metal-oxide, and nanoscale zeolites, combined with microorganisms, provide a sustainable, eco-friendly, and resourceful approach to remediation [33]. Nanoparticles (NPs) are particularly suitable for PAH bioremediation due to their larger surface area, offering abundant active sites for PAH adsorption [34,35]. They immobilize and concentrate PAH molecules through interactions like Van der Waals forces, hydrophobic interactions, and π-π stacking facilitating degradation [36,37]. Some nanomaterials also possess catalytic properties, accelerating PAH breakdown. Yang et al. [38] found that the photocatalytic degradation of FLT in soil was facilitated by a graphitic carbon nitride/iron (III) oxide (g-C3N4/α-Fe2O3) photocatalyst when exposed to sunlight. A dual-doped FeCo/NC catalyst exhibited 24 % better catalytic performance compared to catalysts doped with only Fe or Co, this catalyst was capable of effectively removing 98.87 % of ANT from the soil within 6 h [39]. The interaction between microbes and nanomaterials is complex and varies based on bacterial strain and NPs properties, with mechanisms suggesting bacteria may secrete enzymes immobilized on nanomaterials to aid PAH adsorption and degradation [40]. S-layers and lipopolysaccharides in bacterial cells play a crucial role in NPs attachment and contaminant degradation, affecting biofilm development and microbial colonization. Combining nanomaterials with biochar, enhances bioremediation by increasing porosity and surface area, promoting microbial colonization and PAH adsorption [41,42]. Nanomaterials can also be designed for the controlled release of agents or enzymes, allowing sustained PAH degradation over time. The synergistic effect between nanomaterials and microorganisms improves PAH uptake and degradation, while the easy dispersion of NPs in the environment ensures better distribution and accessibility to contaminated areas.

Most reviews in the scholarly databases focused on either nanomaterial-based remediation or microbe-mediated PAHs remediation, rather than the combined approach of bioremediation and nanomaterial application. Very few articles addressed the integration of these two remediation approaches [37,43,44]. Basak et al. [36] provides the first review offering information on the global use of functionalized NPs for aromatic hydrocarbon remediation. However, none of the reviews provide a clear view on how much the efficiency of PAHs degradation increases while incorporating nanomaterial in bioremediation. There is a lack of systematic reviews, current research compilation, and secondary data analysis on how nanomaterials enhance bioremediation for eliminating PAHs. As such, no strategies for nano-bioremediation of PAHs have been mentioned so far. To overcome this literature gap, this review offers an overview of how the application of nanomaterials influences bioremediation. It also discusses the current research scenario focusing on emerging trends in the use of specific types of PAHs, nanomaterials, and microbes.

The primary objective of the article is to systematically review studies involving both bioremediation and nano-bioremediation of PAHs, discussing the different nanomaterials used and their attributes. It investigates how these nanomaterials influence microbial degradation, thus improving the remediation of PAHs in both liquid and soil environments. Another objective of the study to identify current research trends and international collaborations on this topic. The paper also focuses on different strategies of microbes-mediated nanomaterial for PAHs degradation, highlighting the synergistic effects and mechanisms through which nanomaterials enhance microbial degradation of PAHs. Additionally, it addresses the challenges and future direction involved in using nanomaterials and their implications for remediation.

## 2 PAHs remediation and nanomaterials: A bibliometric analysis

### 2.1 Methodology

**Fig. 2:**
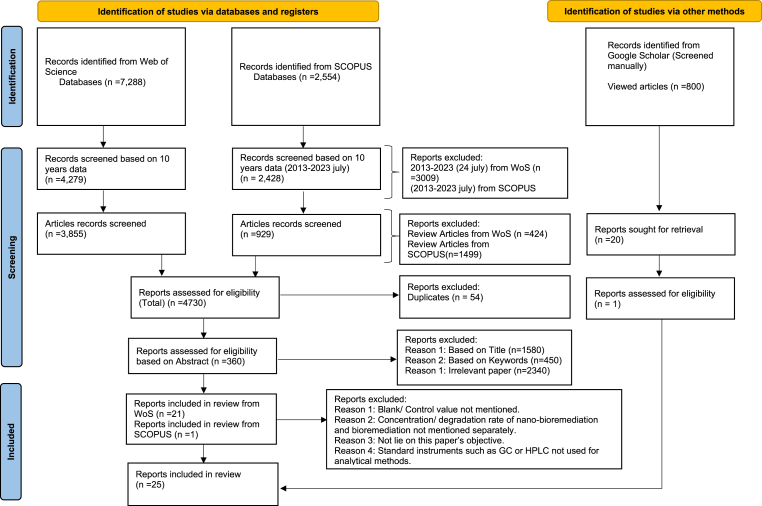
PRISMA flowchart for literature search.

A comprehensive and impartial systematic literature search was conducted using Web of Science, Scopus, and Google Scholar for the keywords “Nanomaterials”, “nanoparticles”, “nanotechnology”, “bioremediation”, “microbial degradation”, “biodegradation”, “Polycyclic Aromatic Hydrocarbons”, and “PAHs” from 2013 to 2023. Boolean search terms combined “AND” logic to widen the scope and “OR” logic within each category to limit irrelevant papers. A total of 4731 articles were exported from the mentioned databases. The review focused on current experimental research, so review papers, book chapters, and duplicates were excluded during the screening process. This filtering resulted in 360 research articles being retained. After reviewing the abstracts and full texts, 26 research publications were selected based on criteria such as the use of standard analytical methodologies and efficient PAH measurement instruments. Additionally, each article included information on the bioremediation and nano-bioremediation PAH degradation percentages for better understanding and analysis. The articles were precisely analyzed for specific data such as the percentage of removal, and the initial and final concentrations of PAHs. If the articles did not directly mention this information but depicted it, the data was extracted directly from the articles by using software like GetData Graphical Digitizer when necessary. The inclusion criteria were directed toward only those datasets that provide a direct comparison between nano-bioremediation and traditional bioremediation under the same experimental conditions. The methodologies and filters used during the literature search are followed by a Prisma flowchart (Fig. 2).

### 2.2 Cooccurrence network

**Fig. 3:**
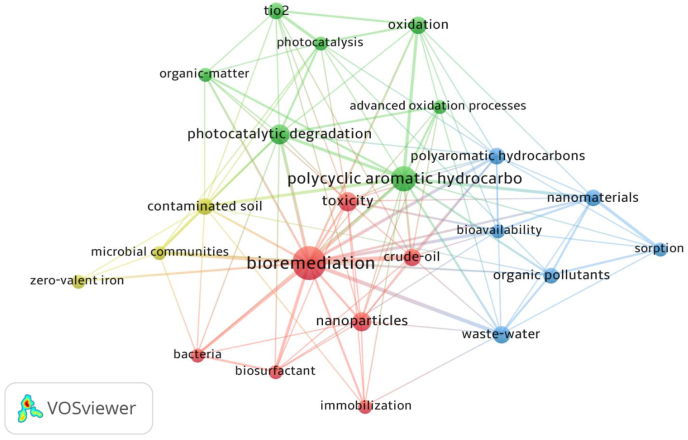
Keywords co-occurrence network analysis.

The exported dataset was utilized to construct a co-occurrence network using VOSviewer software [45], trend topics and country wise contributions were analyzed using the Biblioshiny Package in R Studio version 2023.06.1. Fig. 3 illustrates a fractionalization network generated using VOSviewer software [46,47], where five co-occurrence keywords have been precisely screened and aligned. These keywords represent the most commonly occurring search terms in the research articles analyzed. In this analysis of keyword co-occurrence, a total of four distinct clusters were identified. Varying sizes and colours, highlighting the anecdotal relations, represent the connecting lines between co-occurring keywords and each cluster. Cluster 1 (Red): Bioremediation, sustainable approach employs specific bacterial strains to degrade petroleum hydrocarbons like PAHs found in crude oil. To enhance this process, biosurfactants are produced by these bacteria, which aid in emulsifying the oil, making it more accessible for degradation. Moreover, NPs, such as iron or titanium dioxide (TiO_2_), can be employed in combination with immobilized microbes, enhancing the efficiency of PAH and crude oil degradation. Cluster 2 (Green) Nano-enhanced biodegradation synergistically employs TiO_2_ NPs to catalyze the photocatalytic degradation of PAHs while harnessing bacteria to enhance the degradation of pollutants such as PAHs, crude oil, waste water, and other organic pollutants. Cluster 3 (Blue): Nanomaterials have the potential to substantially alter the bioavailability of organic pollutants like PAHs through enhanced sorption properties, affecting their environmental mobility. Microbes, in turn, play an important role in the biotransformation of these sorbed pollutants, influencing their ultimate fate and environmental impact. Cluster 4 (Yellow): In contaminated or polluted soil, the collaborative act of microbes and NPs such as zero-valent iron initiates the efficient biodegradation of PAHs, offering a sustainable approach to soil remediation.

### 2.3 Trend topics and country-wise contributions

**Fig. 4:**
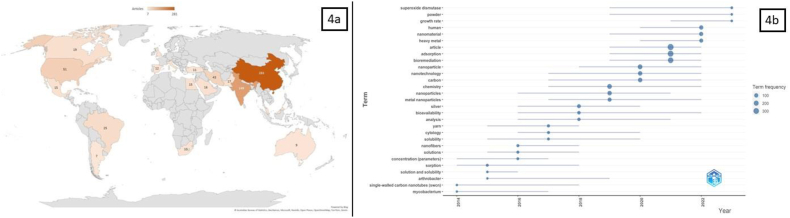
Bibliometric analysis 4a) Global landscape of nano-bioremediation research on PAHs: Country contributions; 4b) Convergence of nanomaterials and bioremediation trends.

Nanomaterial-assisted bioremediation of PAHs and other organic pollutants has emerged as a significant trend within the broader scientific landscape. The data reveals that while nanomaterials and bioremediation were trending topics independently and their convergence gained prominent attention from 2019 to 2022 (high frequency) depicted in Fig. 4a. This synergy offers a promising avenue for addressing the persistent challenge of PAH contamination in the environment. Nanomaterials, including NPs and nanofibers, have shown significant potential for enhancing the performance of bioremediation processes. The porous nanofibers generated through electrospinning cause a substantial surface area that enables the electrostatic attraction of various chemical groups, resulting in the formation of ion exchange membranes. The surface groups of these membranes can be either derived from the polymer or introduced through surface modification. They can be positively, negatively, or both charged [48]. Recent research has been facilitated by advancements in nanotechnology, resulting in the immobilization of microbes or enzymes on electrospun polymeric fibres. PHE, FLT, BAA, and BAP were all removed with efficiencies exceeding 95.1 %, 93.2 %, 79.1 %, and 72.5 % in 6 h, respectively, using laccase carrying electrospun nanofibers. Here, laccase was utilized as the biocatalyst for the degradation of PAHs [49]. By emulsion electrospinning, laccase carrying electrospun fibrous membranes with high laccase catalytic activity and sorption capacity were fabricated, eliminating BAP at 70 % and PHE at 93.8 % from contaminated water. The degradation efficiencies of PAHs by laccase could be noticeably improved by the sorption of PAHs on the laccase-carrying electrospun fibrous membranes, which is higher than free enzyme [50]. These materials can serve as carriers, catalysts, or immobilization matrices for microorganisms, such as *Mycobacterium* or *Arthrobacter*, facilitating their action in degrading PAHs. Additionally, discussions on the bioavailability of pollutants, such as PAHs, have been prominent from 2016 to 2022, highlighting the importance of understanding how nanomaterials can improve the accessibility of these compounds to biodegrading microbes. This trend reflects the global growing interest in developing sustainable and effective remediation techniques for PAH contamination.

Based on the literature search, it was observed that nano-bioremediation of PAHs has gathered significant research attention globally, with China leading to 281 articles (Fig. 4b). India and the USA also contribute significantly with 149 and 51 articles, respectively, highlighting the extensive work on sustainable remediation approaches. Iran, Korea, and other nations like Brazil, Canada, Malaysia, Pakistan, Saudi Arabia, and Egypt have made notable contributions, collectively emphasizing the worldwide interest in harnessing nanotechnology-based bioremediation for PAH remediation.

## 3 Type of nanomaterial

### 3.1 Metallic NPs

Metal NPs, including iron (Fe), silver (Ag), and gold (Au), have received extensive attention in the field of PAH bioremediation due to their considerable surface area and reactivity. These NPs act as carriers for PAH-degrading enzymes or electron mediators, thereby aiding in PAH degradation. Apart from metallic NPs, various other nanomaterials, such as photocatalytic nanomaterials, biopolymers, and CNTs, have also been explored for their potential to address PAH contamination [41]. NPs have proven effective in breaking down PAHs in both water and soil environments. In the study by Al-Zaban et al. [51] study, indigenous fungi, specifically *Aspergillus flavus* and *Trichoderma harzianum*, were employed for *in situ* crude oil bioremediation by employing silver NPs (AgNPs). As much as 57.8 % crude oil degradation was achieved within one week of incubation under optimum conditions at 30 °C, pH 7, 4 g/L of crude oil concentration, 0.05g of AgNPs, and two fungal strains with equal proportion. This demonstrates the potential of low concentrations of NPs in enhancing the biodegradation process, emphasizing the role of nanomaterials in environmental remediation.

### 3.2 Metal oxide NPs

Metal oxide NPs (e.g. Fe_3_O_4_, ZnO, and TiO_2_) exhibit potent photocatalytic properties, enabling the degradation of PAHs when exposed to light. These nanomaterials effectively break down PAHs within the aquatic and soil environments [52]. Innovative 3D-printed photocatalyst-polymer composites are also promising for PAH degradation in complex mixtures [53]. The microbial strain *B*. *licheniformis* showed its bioremediation efficiency to crude oil especially when combined with biosurfactant and NPs (Zn_5_(OH)_8_Cl_2_ at 0.1 g/100 ml concentration or Fe_2_O_3_ at 0.2g/100 ml concentration), was 60 % of crude oil [54]. Oyewole et al. [55] observed that *Alcaligenes faecalis*, in combination with iron oxide NPs and biosurfactants, exhibited increased crude oil biodegradation of up to 84 % of petroleum from contaminated soil. Biosurfactants prevented NPs oxidation and aggregation, enhancing their reactivity for petroleum remediation. According to Parthipan et al. [56], the iron NPs (Fe_3_O_4_) were employed to enhance the mineralization process of blend PAHs (PYR, ANT and BAP) by a bacterial consortium composed of *Pseudomonas stutzeri* and *Acinetobacter baumannii*. An integrated approach, involving *B*. *subtilis* producing biosurfactants, iron NPs, and the bacterial consortium led to an impressive 85 % degradation of mixed PAHs. The inclusion of iron NPs not only enhanced microbial biomass but also facilitated the adsorption of PAHs, ultimately contributing to the proficient removal of these complicated contaminants found in soil and aquatic surroundings.

### 3.3 Carbon-based materials (CBMs)

CBMs are composed of sp2-hybridized carbon atoms, exhibiting multi-dimensional hybridization. CBMs, such as CNTs, carbon quantum dots (CQDs), carbon black, graphene oxide, and graphene, constitute a versatile class of nanomaterials well-known for their proficiency in PAH adsorption [44]. These nanomaterials could be further improved by incorporating functional groups like carboxyl groups, hydroxyl, and carboxylic acid, thereby imparting hydrophilic properties. This modification enables them to effectively sorb polar compounds with relatively lower molecular weights [57]. CQDs are an advanced carbon nanomaterial that are universally employed in a variety of disciplines due to their exceptional properties. CQDs have been employed to enhance the electronic transmission capabilities of binary semiconductor nanomaterials [58]. Fluorescence, water solubility, biocompatibility, low toxicity, small size and ease of modification, inexpensive scale-up production, and versatile conjugation with other NPs are among the numerous advantageous properties of CQDs [59]. CQDs utilizes selective absorption wavelength for PAHs elimination [60]. *Labedella gwakjiensis* demonstrated potent PAHs biodegradation capabilities in saline oil-contaminated soils. With the addition of carbon quantum dots iron oxide (CQD.Fe_3_O_4_) NPs at a concentration of 0.5 g/L significantly enhanced PAHs degradation, particularly PHE, with a degradation rate of 63.63 % and 81.77 % after 48 and 72 h, respectively [61]. The use of graphene-based NPs for PHE extraction has proven to be successful reported by Zhao et al. [62]. Mahpishanian et al. [63] effectively utilized a composite of graphene oxide nanosheets and silica-coated Fe_3_O_4_ microparticles, modified with 2-phenylethylamine, for the isolation of a variety of PAHs from aqueous solutions. The hybrid nanostructure formed by gold NPs immobilized in laccase enzyme (laccase-AuNPs@vesicles) achieves a 98.5 % reduction in 4-nitrophenol, surpassing free laccase efficiency by 2.3 times, indicating its superior catalytic performance in environmental remediation [64]. Graphene oxide has proven effective as the substrate for immobilizing bacteria, offering protection in intricate soil environments and enhancing their capabilities in addressing hydrocarbons polluted sites [65]. CNTs, such as single-walled CNTs (SWCNTs) and multiwalled CNTs (MWCNTs) are valued for their chemical stability, thermal resistance, strong adsorption, pH tolerance, and π-π and Van der Waals interactions with PAHs making them excellent adsorbents for contaminant removal [66].

### 3.4 Magnetic NPs

A magnetic adsorbent was created by blending Fe_3_O_4_ NPs, MWCNTs, and polypyrrole. Interactions between π–π were crucial, facilitated by CNTs and polypyrrole's π bonds. Polypyrrole's –NH_2_ groups aided material dispersion in water. Versatile iron and carbon composites are also promising. NPs like ZnFe_2_O_4_ were enclosed in a carbon matrix for superparamagnetic C/ZnFe_2_O_4_. The nanocomposite effectively removed NAP and 2-naphthol from water, driven by electrostatic interactions between PAHs and C/ZnFe_2_O_4_ for efficient pollutant remediation [67]. Magnetic NPs are a potential agent for environmental remediation facilitated by huge surface area, magnetic receptiveness, and affluence of functionalization [68]. Utilization of magnetic chromium ferrite (CrFe_2_O_4_) NPs aid in the degradation of ANT, PHE and NAP up to 99 %, 90 %, and 86 % respectively [69].

### 3.5 Nanocomposites

Composites are solid materials with at least one phase <100 nm. Combining metallic NPs and nano supports creates nanocomposites, boosting surface area and adsorption. They protect microorganisms, preventing degradation by toxins, making them ideal for removing pollutants. Magnetic activated carbon nanocomposite from green tea leaf waste effectively removed PAHs, adsorbing them at rates of 28.08, 22.75, 19.14, and 15.86 mg/g for BBF, BAP, CHRY, and BAA respectively [70]. In another study, Mukwevho et al. [71] synthesized a ZnO/Ag/GO nanocomposite, preserving ZnO hexagonal structure. That nanomaterial demonstrated a notable adsorption capacity of 500 mg/g for NAP removal. Through photodegradation, it achieved an 80 % reduction in NAP within 20 min, with further enhancement to 92 % reduction in 50 min. Rani et al. [72] reported that iron oxide based chitosan nanocomposites, specifically ZnFe₂O₄-CS, achieved degradation efficiencies of 95 % for ANT and 92 % for PHE. The NiO–ZnO bimetallic oxide nanocomposites able to degrade 2 ppm of PAHs, achieving 98 % degradation of ANT and 93 % of PHE within 12 h under sunlight exposure [73]. TiO_2_ based zinc hexacyanoferrate framework (TiO_2_@ZnHCF) nanocomposite demonstrated superior photocatalytic degradation of PAHs, achieving removal rates of 93%–96 % in water, 82%–86 % in soil, and 81.63%–85.43 % in river sediment [73]. The bacterial consortium of *Flavobacterium johnsoniae* and *Shewanella baltica*, immobilized on a goethite-chitosan nanocomposite, achieved a maximum PAHs degradation efficiency of 93.32 % within 3 days [54]. Bioremediation of crude oil in polluted surface water can be effectively achieved using specialized alginate-based nanocomposite beads containing iron oxide NPs immobilized with *Bacillus, Pseudomonas*, and *Klebsiella pneumoniae* on biochar, able to degrade 93.7 % of PAHs [74]. This nanocomposite shows promise for efficient PAHs degradation.

### 3.6 Metal-organic frameworks (MOFs)

MOFs are highly specialized nanomaterials characterized by their unique porous and crystalline structure, created through the coordination of metal ions or clusters with organic ligands. MOFs are renowned for their exceptional surface area and customizable porosity, reusability, and versatile design options, making them invaluable for gas storage, separation, catalysis, and drug delivery applications. These materials offer a precisely engineered structure, allowing them to be tailored for specific uses. MOFs, along with other advanced nanosorbents like nano-polymers, represent the forefront of petroleum wastewater treatment, showcasing their potential in the efficient removal of pollutants like PAHs [75,76]. Using bimetallic metal-organic frameworks with 1,4-benzenedicarboxylic acid activated through peroxymonosulfate, 99 % of PHE removal efficiency was reported at pH 3.15, 1.0 mg/L PHE, and a reaction time of 30 min [77]. While MOFs offer benefits like low energy consumption and high efficiency, it often suffers from instability in aqueous environments, leading to phase changes, loss of crystallinity, and structural decomposition. Research suggests that incorporating secondary metal nodes can enhance both catalytic activity and stability, resulting in bimetallic MOFs. Peroxymonosulfate activated molecularly imprinted bimetallic MOFs (Al/Co-MOFs@MIP) effectively target the removal of PAHs from soil washing effluents, resulting in 94 % PHE degradation in 1 h 30 min [78]. The study found that using nanomaterials like zeolite imidazolate framework-8 (ZIF-8) and combining it with citric acid (CA) greatly improved the removal of PHE by *B*. *subtilis*. The integration of ZIF-8 and CA significantly improved the growth and cell viability of *Bacillus subtilis* ZL09–26, while also reducing the toxic effects of PHE stress. Acting as an anionic surfactant, CA modified the surface charge of ZIF-8, facilitating the formation of a biomimetic mineralized protective shell around the bacteria. This ZIF-8-CA coating, characterized by surface roughening and particle aggregation, effectively encapsulated the bacteria, enhancing their ability to degrade PAHs. Compared to the control condition (*Bacillus subtilis* ZL09–26 alone), these nano-modified conditions showed significant increases in PHE removal rates. Particularly, *Bacillus subtilis* ZL09–26@ZIF-8-CA was highly influential, removing 94.14 % of PHE in just 6 days. These highlights are the vital character of the nanomaterials in enhancing PAHs cleanup when combined with bacteria [79].

## 4 Nanomaterial attributes facilitating PAH removal

Recently, as an emerging technology, interest growing in utilizing nanomaterial-based tools for practical solutions for the remediation of various pollutants in contaminated sites. In this regard, a wide array of nanomaterials has been introduced into the market as nanosorbents, offering enhanced capabilities for treating water contaminated with PAHs, ultimately making it suitable for reclaim [75]. Nanomaterials possess structural elements with dimensions ranging from 1 to 100 nm (nm) in at least one dimension. These materials stand out due to their unique characteristics, notably a significantly increased surface-to-volume ratio as well as enhanced magnetic and catalytic traits when compared to their bulk materials [80].

Nanomaterials are characterized by their diminutive dimension, which enables them to have a greater surface-to-volume ratio. This attribute allows them to engage in more significant interactions with the molecules in their environment. As the dimension decreases, the concentration of ions on the surface increases, thereby increasing their reactivity. Nanomaterials possess distinctive characteristics that render them highly desirable in various applications, such as catalysis and sensing [81,82]. In many cases, nanomaterials exhibit distinctive magnetic properties. By treating hydrocarbon-degrading microbial cells like *Alcanivorax borkumensis* having a positively charged polymer i.e., polyallylamine hydrochloride and layered magnetic nanomaterials, a protective shell of about 70–100 nm was formed on the cell wall. Polycation coated magnetic NPs utilize the direct deposition of positively charged iron oxide NPs onto microbial cells during brief incubation in high NP concentrations. Gram-negative bacteria have cell walls with a thin peptidoglycan layer between the outer membrane and inner plasma membrane. The presence of lipopolysaccharides gives the cell walls a negative charge, attracting cationic particles through electrostatic interactions. These intact cells exhibit a negative potential of −16 mV, facilitating the rapid deposition of cationic magnetic complexes on the bacterial cell walls. A cationic charge of polyallylamine and 20 nm iron oxide NPs enables a swift, single-step encapsulation process by exploiting electrostatic interactions with bacterial surfaces. The cationic polycation-coated magnetic NPs act as electron donors, while the negatively charged bacterial cell walls serve as electron acceptors. This electrostatic interaction facilitates the rapid deposition of cationic magnetic complexes on the bacterial surfaces, forming a charge transfer complex [83]. This innovative approach enhances the efficacy and endurance of cells in the degradation of hydrocarbons. The extensive surface area of nanomaterials leads to a highly active reaction environment, which in turn enhances their catalytic efficacy. Quantum size effects at the nanoscale enhance the efficacy of catalytic processes by modifying electronic structures. Nano-catalysts enhance reaction selectivity at lower temperatures, reduce side reactions, promote recycling, and reduce environmental and health risks. These advances in catalytic nanotechnology promote greener and more sustainable processes by replacing low-quality materials with NPs [84]. Immobilized microbial cells not only improve process stability and catalytic efficiency but also simplify the cell recovery for subsequent reuse. Enzymes can be fixed onto NPs via methods such as adsorption, entrapment, covalent bonding, or membrane confinement. Immobilized enzymes exhibited approximately a twofold increase in catalytic activity compared to their native counterparts, primarily due to heightened surface hydrophobic nature. Their stability depends on the number of bonds formed between the NPs and enzymes [85]. Acevedo et al. [86] harnessed manganese peroxidase (MnP) from the chilean white-rot fungus *Anthracophyllum discolor*, immobilizing it onto nano clay (100 nm) derived from volcanic soil. Interestingly, physical adsorption process successfully immobilized 75 % of the enzyme. The immobilized MnP demonstrated superior PAH degradation, particularly with PYR (>86 %) and ANT (>65 %) individually. It also exhibited some capacity to degrade FLT (<15.2 %) and PHE (<8.6 %).

Nanomaterials possess remarkable mechanical properties, including high strength and flexibility. This is attributed to an increased density of defects and interfaces in nanoscale structures. CNTs and graphene are especially well-known for their exceptional mechanical strength, with applications in nanocomposites [[87], [88], [89]]. Combining crude enzymes from *Trametes maxima* and *Paecelomices carneous* within alginate beads and trimetallic TiO_2_–C–Ag NPs enhances their mechanical stability and resistance to protease attack. This hybrid nanomaterial exhibits increased PHE removal capacity, achieving 94.3 % removal in continuous mode [90,91]. Nanomaterials possess distinct electrical conductivity properties due to their size and unique characteristics, differing from macroscopic materials. However, they generally exhibit lower thermal and electrical conductivity than bulk materials.

## 5 Bioremediation and nano-bioremediation for PAHs degradation in liquid and soil samples

Table 1, Table 2 provide a comparative analysis of nano-bioremediation and bioremediation for PAHs remediation under the same experimental design, specific to liquid and soil samples, respectively. The compilation of data extracted from 26 current research papers focusing on the assessment of bioremediation rates, initial and final concentrations of PAHs, as well as nano-bioremediation rates, type of nanomaterial used initial and final concentrations, and various other parameters (Tables S1 and S2). Zhoa et al. [93] reported that reduced graphene oxide demonstrated a 99 % degradation of NAP in nano-bioremediation with *Burkholderia cepacia,* compared to 67.3 % with bioremediation alone. Therefore, addition of nanomaterial in bioremediation increased the PAHs remediation. The degradation condition for ANT was achieved using titanium dioxide NPs with *Alcaligenes faecalis* in a nano-bioremediation approach, resulting in a 37.9 % degradation, compared to 24.2 % with *Alcaligenes faecalis* [43]. In bioremediation (phytoremediation) without NPs, Proteobacteria, Actinobacteria, Bacteroidota, and Firmicutes achieved a 55.5 % degradation of BBF. Using graphene oxide in a nano-bioremediation approach with the same microbial consortia, the degradation of BBF increased to 74.22 % [107]. Chai et al. [110] reported that bioremediation using lignin peroxidase (LiP)-extracted from *Pichia methanolica* targeting PHE achieved 23.7 % efficiency, whereas nano-bioremediation with chitosan-modified halloysite nanotubes and *Pichia methanolica* (LiP) targeting PHE achieved approximately double the removal efficiency at 51.3 %.

**Table 1:** Bioremediation and Nano-Bioremediation for PAHs Degradation in Liquid samples.

Remediation method	Type of NP	Bioagent	PAHs type	Size of NP (nm)	Shape of NP	PAHs degradation (%)	References
Bioremediation	Without NP	*Labedella gwakjiensis*	PHE	NA	NA	37.12	[61]
Nano-Bioremediation	Carbon Quantum Dots conjugated with Iron (III) Oxide	*Labedella gwakjiensis*	PHE	10	Spherical	81.77	

Bioremediation	Without NP	*Alcaligenes faecalis*	ANT	NA	NA	10.4	[43]
Nano-Bioremediation	Titanium Dioxide NPs	*Alcaligenes faecalis*	ANT	17.11	Spherical and granular	21.3	

Bioremediation	Without NP	*Achromobacter* sp.	PHE	NA	NA	56.24	[92]
Nano-Bioremediation	Titanium Dioxide with biochar	*Achromobacter* sp.	PHE	276.1	Stacked graphite sheet structure and rectangular shaped	72.58	

Bioremediation	Without NP	*Burkholderia cepacia*	NAP	NA	NA	67.3	[93]
Nano-Bioremediation	Reduced graphene oxide	*Burkholderia cepacia*	NAP	NM	NM	99.0	

Bioremediation	Without NP	*Pseudomonas* sp. *Rhodocucus* sp.	ANT	NA	NA	58.3	[94]
Nano-Bioremediation	Polyimide aerogels	*Pseudomonas* sp. *Rhodocucus* sp.	ANT	NM	NM	78.6	

Bioremediation	Without NP	*Pseudomonas stutzeri* and *Acinetobacter baumannii*	ANT, PYR, BAP	NA	NA	52.6	[56]
Nano-Bioremediation	Iron nanoparticles	*Pseudomonas stutzeri* and *Acinetobacter baumannii*	ANT, PYR, BAP	134	Spherical	65.7	
Nano-Bioremediation	Iron nanoparticles Biosurfactant (*Bacillus subtilis*)	*Pseudomonas stutzeri* and *Acinetobacter baumannii*	ANT, PYR, BAP	134	Spherical	85	

Bioremediation	Without NP	*Penicillium oxalicum*	PYR	NA	NA	72	[95]
Nano-Bioremediation	Carbon nanotube composites	*Penicillium oxalicum*	PYR	NM	NM	90	

Bioremediation	Without NP	Biofilm from consortia Planococcaceae, Oxalobacteraceae etc	PHE	NA	NA	12.97 ± 0.44	[96]
Nano-Bioremediation	Copper and Nitrogen co-doped Titanium Dioxide	Biofilm from consortia Planococcaceae, Oxalobacteraceae etc	PHE	10–30	NM	88.63	

Bioremediation	Without NP	Biofilm from consortia Planococcaceae, Oxalobacteraceae etc	PYR	NA	NA	6.65	[97]
Nano-Bioremediation	Copper and Nitrogen co-doped Titanium Dioxide	Biofilm from consortia Planococcaceae, Oxalobacteraceae etc	PYR	10–30	NM	63.89	

Bioremediation	Without NP	*Candida tropicalis*	ICP	NA	NA	61	[98]
Nano-Bioremediation	Iron nanoparticles	*Candida tropicalis*	ICP	50	Spherical	75	

Bioremediation	Without NP	*Rhodotorula* sp. *Debaryomyces hansenii* and *Hanseniaspora valbyensis*	BghiP	NA	NA	60.0	[99]
Nano-Bioremediation	Zinc Oxide	*Rhodotorula* sp. *Debaryomyces hansenii* and *Hanseniaspora valbyensis*	BghiP	10	Rod	60.7	

Bioremediation	Without NP	*Rhodotorula* sp. *Hanseniaspora opuntiae* and *Debaryomyces hansenii*	BAP	NA	NA	76.0	[100]
Nano-Bioremediation	Zinc Oxide	*Rhodotorula* sp. *Hanseniaspora opuntiae* and *Debaryomyces hansenii*	BAP	50	Spherical	77.2	

Bioremediation	Without NP	*Bacillus thuringiensis*	PHE	NA	NA	65.71	[101]
Nano-Bioremediation	Multi-Walled Carbon Nanotube Buckypaper	*Bacillus thuringiensis*	PHE	200	Spherical	93.81	

Bioremediation	Without NP	*Sphingomonas* sp.	PHE	NA	NA	74.6	[102]
Nano-Bioremediation	Nano bamboo charcoal	*Sphingomonas* sp.	PHE	NM	NM	93.01	

Bioremediation	Without NP	*Paracoccus* sp.	BAP	NA	NA	60	[103]
Nano-Bioremediation	Hematite NPs	*Paracoccus* sp.	BAP	NM	NM	45.8	

Bioremediation	Without NP	*Sphingomonas* sp.	PHE	NA	NA	69.83	[104]
Nano-Bioremediation	Nano bamboo charcoal	*Sphingomonas* sp.	PHE	401.9	Irregular	94	

Bioremediation	Without NP	Methanosarcina and Methanosaeta, Pseudomonas, Cloastridia, and Synergistetes	PYR	NA	NA	40.8	[105]
Nano-Bioremediation	Iron (II) Sulfide	Methanosarcina and Methanosaeta, Pseudomonas, Cloastridia, and Synergistetes	PYR	20–50	NM	77.5	
Nano-Bioremediation	Magnetic carbon	Methanosarcina and Methanosaeta, Pseudomonas, Cloastridia, and Synergistetes	PYR	20–50	NM	72.1	

Bioremediation	Without NP	Archaea and methanogen	PHE	NA	NA	60.52	[106]
Nano-Bioremediation	Magnetite powder	Archaea and methanogen	PHE	50–100	Spherical	70.94	
Nano-Bioremediation	Nanoscale Iron (III) Oxide	Archaea and methanogen	PHE	50–100	Spherical	70.89	

NA=Not Applicable, NM=Not Mentioned.

**Table 2:** Bioremediation and Nano-Bioremediation for PAHs Degradation in soil sample.

Remediation method	Type of NPs	Bioagent	PAHs type	Size of NPs (nm)	Shape of NPs	PAHs degradation (%)	References
Bioremediation	Without NP	*Alcaligenes faecalis*	ANT	NA	NA	24.2	[43]
Nano-Bioremediation	Titanium Dioxide NPs	*Alcaligenes faecalis*	ANT	17.11	Spherical and granular	37.9	

Bioremediation (phytoremediation)	Without NP	Proteobacteria, Actinobacteria, Bacteroidota,Firmicutes etc and Fire Phoenix (Plant Species)	FLT	NA	NA	92.60	[107]
Nano-Bioremediation	Graphene oxide	Proteobacteria, Actinobacteria, Bacteroidota,Firmicutes etc and Fire Phoenix (Plant Species)	FLT	NM	NM	95.57	

Bioremediation (phytoremediation)	Without NP	Proteobacteria, Actinobacteria, Bacteroidota,Firmicutes etc and Fire Phoenix (Plant Species)	PYR	NA	NA	90.11	[107]
Nano-Bioremediation	Graphene oxide	Proteobacteria, Actinobacteria, Bacteroidota,Firmicutes etc and Fire Phoenix (Plant Species)	PYR	NM	NM	95.88	

Bioremediation (phytoremediation)	Without NP	Proteobacteria, Actinobacteria, Bacteroidota,Firmicutes etc and Fire Phoenix (Plant Species)	BAA	NA	NA	87.70	[107]
Nano-Bioremediation	Graphene oxide	Proteobacteria, Actinobacteria, Bacteroidota,Firmicutes etc and Fire Phoenix (Plant Species)	BAA	NM	NM	93.30	

Bioremediation (phytoremediation)	Without NP	Proteobacteria, Actinobacteria, Bacteroidota,Firmicutes etc and Fire Phoenix (Plant Species)	CHRY	NA	NA	88.85	[107]
Nano-Bioremediation	Graphene oxide	Proteobacteria, Actinobacteria, Bacteroidota,Firmicutes etc and Fire Phoenix (Plant Species)	CHRY	NM	NM	91.82	

Bioremediation (phytoremediation)	Without NP	Proteobacteria, Actinobacteria, Bacteroidota,Firmicutes etc and Fire Phoenix (Plant Species)	BBF	NA	NA	55.50	[107]
Nano-Bioremediation	Graphene oxide	Proteobacteria, Actinobacteria, Bacteroidota,Firmicutes etc and Fire Phoenix (Plant Species)	BBF	NM	NM	74.22	

Bioremediation (phytoremediation)	Without NP	Proteobacteria, Actinobacteria, Bacteroidota,Firmicutes etc and Fire Phoenix (Plant Species)	BKF	NA	NA	55.97	[107]
Nano-Bioremediation	Graphene oxide	Proteobacteria, Actinobacteria, Bacteroidota,Firmicutes etc and Fire Phoenix (Plant Species)	BKF	NM	NM	62.00	

Bioremediation (phytoremediation)	Without NP	Proteobacteria, Actinobacteria, Bacteroidota,Firmicutes etc and Fire Phoenix (Plant Species)	BAP	NA	NA	56.15	[107]
Nano-Bioremediation	Graphene oxide	Proteobacteria, Actinobacteria, Bacteroidota,Firmicutes etc and Fire Phoenix (Plant Species)	BAP	NM	NM	67.37	

Bioremediation (phytoremediation)	Without NP	Proteobacteria, Actinobacteria, Bacteroidota,Firmicutes etc and Fire Phoenix (Plant Species)	DBA	NA	NA	58.03	[107]
Nano-Bioremediation	Graphene oxide	Proteobacteria, Actinobacteria, Bacteroidota,Firmicutes etc and Fire Phoenix (Plant Species)	DBA	NM	NM	67.75	

Bioremediation	Without NP	Proteobacteria, Acidobacteria, Gemmatimonadota, Bacteroidota (Soil microbes)	PAHs	NA	NA	19.57	[108]
Nano-Bioremediation	Graphene oxide	Proteobacteria, Acidobacteria, Gemmatimonadota, Bacteroidota (Soil microbes)	PAHs	NM	Irregular	41.07	

Bioremediation	Without NP	*Geobacter* and *Geothrix*	PAHs	NA	NA	8.7	[109]
Nano-Bioremediation	Magnetite NPs	*Geobacter* and *Geothrix*	PAHs	100	Spherical	86	

Bioremediation	Without NP	*Pichia methanolica* (LiP)	PHE	NA	NA	23.7	[110]
Nano-Bioremediation	Chitosan-modified halloysite nanotubes	*Pichia methanolica* (LiP)	PHE	NM	Rod	51.3	

Bioremediation	Without NP	*Pichia methanolica* (LiP)	FLU	NA	NA	25	[110]
Nano-Bioremediation	Chitosan-modified halloysite nanotubes	*Pichia methanolica* (LiP)	FLU	NM	Rod	38.1	

Bioremediation	Without NP	Soil Microbes	NAP	NA	NA	92.7	[111]
Nano-Bioremediation	Silver Phosphate on Iron (III) Oxide NP	Soil Microbes	NAP	20–40	Irregular	93.7	

Bioremediation	Without NP	Soil Microbes	ANT	NA	NA	84.8	[111]
Nano-Bioremediation	Silver Phosphate on Iron (III) Oxide NP	Soil Microbes	ANT	20–40	Irregular	91.9	

Bioremediation	Without NP	Soil Microbes	PHE	NA	NA	86	[111]
Nano-Bioremediation	Silver Phosphate on Iron (III) Oxide NP	Soil Microbes	PHE	20–40	Irregular	94.6	

Bioremediation	Without NP	Soil Microbes	FLU	NA	NA	68.6	[111]
Nano-Bioremediation	Silver Phosphate on Iron (III) Oxide NP	Soil Microbes	FLU	20–40	Irregular	82.3	

Bioremediation	Without NP	Soil Microbes	PYR	NA	NA	63.4	[111]
Nano-Bioremediation	Silver Phosphate on Iron (III) Oxide NP	Soil Microbes	PYR	20–40	Irregular	78.8	

Bioremediation	Without NP	*Paracoccus aminovorans* + Soil microbes	PAHs	NA	NA	46.9	[65]
Nano-Bioremediation	Graphene oxide	*Paracoccus aminovorans* + Soil microbes	PAHs	17.92	Irregular	62.86	

Bioremediation	Without NP	*Bacillus cereus, Acidovorax wohlfahrtii,* and *Bacillus thuringiensis*	PYR	NA	NA	88	[112]
Nano-Bioremediation	Hematite NPs	*Bacillus cereus, Acidovorax wohlfahrtii,* and *Bacillus thuringiensis*	PYR	28–55	Spherical	96	

NA=Not Applicable, NM=Not Mentioned.

### 5.1 Correlation matrix

**Fig. 5:**
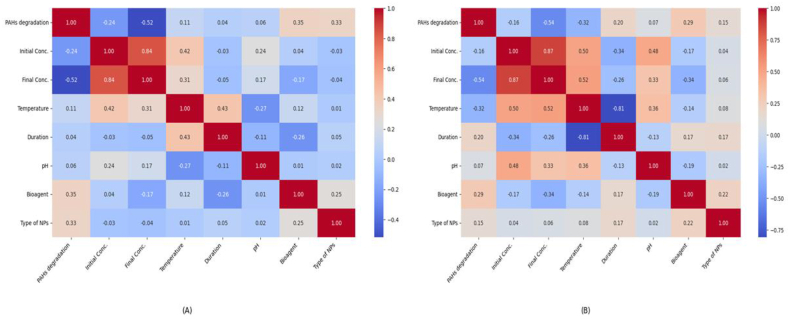
Correlation matrix heatmap for A) Liquid samples B) Soil samples.

Fig. 5a and b shows that the degradation of PAHs effectively lowers their final concentration, since there is a negative link between degradation and concentration in both soil and liquid samples as per different studies cited in Supplementary Tables S1 and S2. A moderate positive association was seen between the presence of bioagents and the degradation of PAHs in both the soil and liquid samples, suggesting that specific bioagents can accelerate degradation in these settings. In contrast, soil samples show a moderate positive correlation with duration and a negative correlation with pH, indicating that higher duration may extend the degradation process and lower pH levels, respectively. Nevertheless, there is a strong negative correlation of temperature, showing that higher temperatures considerably shorten the degradation duration. Liquid samples reveal that the type of NPs affirms a stronger positive correlation with PAHs degradation and bioagent, indicating that NPs play a substantial role in both liquid and soil medium for improving degradation and interacting with bioagents. Temperature significantly influences PAH breakdown in soil samples, time and bioagents exert a minor influence whereas the effects of pH and NP type are minimal. Temperature impacts duration and pH in liquid samples, while bioagents are more important in solid samples. When compared to soil samples, NP type has a more significant impact on bioagent interaction and degradation.

### 5.2 Size and shape of NPs

The small size of nanomaterials provides with high surface area and are strongly adsorbed on the surfaces of microbial cells. Additionally, electrostatic interactions are a crucial factor of the interacting force between cells and NPs [113]. The desirable modifications in size and shape of nanomaterials improve its functioning, which may offer important advantage for elimination of contaminants [114,115]. On reviewing, it was generally found that the most frequent sizes of NPs were 40 nm and 50 nm. No studies utilized particles smaller than 10 nm due to their ability to penetrate inside cells, although some involving complex structures exhibited a wider range of nano-sizes. As per the literature, most common shape for nanomaterial was spherical. Spherical shapes often enhance mobility and stability, while irregular shapes may offer more active sites for adsorption. Spherical nanomaterials tend to aggregate less, enhancing their effectiveness in nano-bioremediation. Different shapes of nanomaterials affect their surface area, reactivity, and interaction with contaminants and microorganisms in bioremediation [116].

Biosynthesized palladium NPs, due to their smaller size and higher surface-to-volume ratio, validated superior catalytic performance compared to chemically reduced palladium [117]. Its small size allows it to penetrate deep into polluted regions that microbes cannot reach, thereby extending the applications of nano-bioremediation. Therefore, it is crucial to understand the interactions between NPs and microbes. However, the small size of NPs presents both advantages and limitations. Less soluble NPs, such as gold, platinum, and silver, are generally less toxic compared to others. NPs sized 1–12 nm can enter microbial cells, stimulating the production of reactive oxygen species and thereby reducing microbial growth [118]. El Bestawy et al. [113] found that NPs (10–20 nm) penetrated cells causing cell destruction when incubated for 6 h. To prevent the toxicity, the experiment runs for 4 h creating effective Fe₃O₄-immobilized bacterial cultures capable of degrading total petroleum hydrocarbons by 85 %. Therefore, research should be focused on optimizing methods to avoid NPs toxicity in soil and water, understanding their interactions with biotic and abiotic agents.

### 5.3 Bioremediation versus nano-bioremediation

**Fig. 6:**
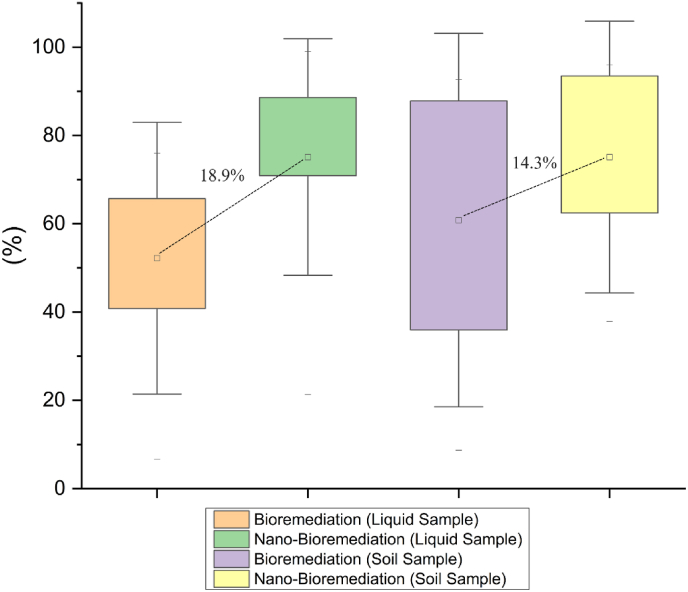
Different attributes of nano-bioremediation facilitating PAHs breakdown.

The compilation of collected secondary data, irrespective of types of PAHs, microbial strains, nanomaterial types, concentrations, and other experimental setups, yielded a generalized outcome, and its graphical representation is illustrated in Fig. 6. It was observed that, when compared, traditional bioremediation achieved an average removal efficiency of 52.2 %, while nano-bioremediation exhibited a higher average removal efficiency of 71.1 %. The application of NPs in bioremediation greatly enhanced the efficiency of remediation, resulting in an 18.9 % increase in the elimination of PAHs in liquid samples. Soil sample analysis consistently revealed that traditional bioremediation attained an average removal efficiency of 60.8 %. However, when nano-bioremediation is included, there is a significant increase of 14.3 %, resulting in an average removal efficiency of 75.1 %. The pattern emphasised the exceptional hybrid capabilities of nano-bioremediation in effectively eliminating PAHs from the environment.

## 6 Nanomaterial-aided microbial PAH degradation strategies

Nano-bioremediation technology offers a potential remedial approach for PAH in contaminated soil, contributing to environmental welfare. Its application enhances water and soil quality, nurturing agricultural productivity, vegetation growth, and microbial survival [41]. An additional advantage is its ability to prevent hazardous compounds from leaching into groundwater [14]. The interaction between NPs functional surfaces and biological interfaces enhances environmental restoration. Emerging NPs types and concentrations provide a solution for recalcitrant PAH biodegradation [34,41]. Previous research highlights specific roles of NPs in enhancing biodegradation rates. For a decade, environmental specialists have focused on remediating PAHs at contaminated sites, facing multifaceted challenges such as soil properties, secondary pollution, treatment duration, and budget constraints. Physicochemical and biological remediation methods, like incineration, electrokinetic, soil washing, and phytoremediation, have been explored [1,29,119]. Physicochemical approaches involve limitations such as high costs and monitoring complications [120]. In contrast, integrating nanotechnology and biological methods offers an environmentally friendly approach to addressing the environmental impact of PAHs contamination.

**Fig. 7:**
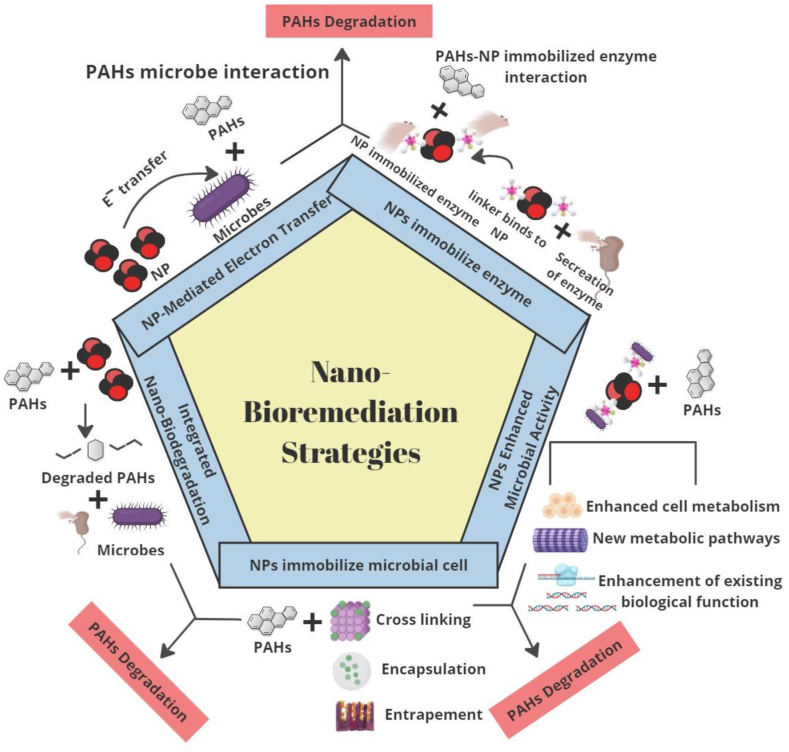
Nano-bioremediation strategies for enhanced PAH degradation.

The interaction between microbes and NPs in the adsorption of PAHs is complicated, mainly attributed to the complex molecular structures of PAHs. Mechanistic insights into this interaction remain limited, and studies assuming these mechanisms are not yet fully elucidated. Numerous research endeavors have attempted to elucidate the potential degradation strategies of PAHs, which exhibit variation across microbial strains and NP characteristics [54,56]. The review explores various strategies for nano-bioremediation aimed at the breakdown of PAHs. Fig. 7 illustrates the key mechanisms of nano-bioremediation strategies for PAH degradation. Nanoparticle-mediated electron transfer demonstrates the transfer of electrons from NP to microbes, initiating reactions that lead to the degradation of PAHs. Enzyme-immobilized NP, where microbes secrete enzymes that are immobilized onto NPs through linkers, enabling effective interactions for PAH breakdown. NP enhances microbial activity by promoting enhanced cell metabolism and new metabolic pathways that contribute to the degradation of PAHs. The microbial cell immobilized NPs, employ techniques such as cross-linking, entrapment, and encapsulation for efficient immobilization and targeted PAH biodegradation. Lastly, integrated nano-bioremediation is mentioned, emphasizing the combined action of nanomaterials and biodegradation pathways for comprehensive PAH remediation.

### 6.1 Microbial cell immobilized NPs

NPs offer an advanced means of immobilizing microbial cells or consortia for targeted chemical degradation or reclamation [113]. Immobilization confines microbial cells or enzymes within a solid space, preserving their catalytic activity for reuse. Techniques include cross-linkage, encapsulation, adsorption, and entrapment. Surface-confined microbes exhibit resistance to harsh conditions, enhanced stability, greater biomass, and improved contaminant degradation potential [121]. In contrast to conventional methods involving micron-sized media or fixed surfaces, magnetic NPs like Fe_3_O_4_, functionalized with ammonium oleate, have been applied to coat *P*. *delafieldi* [122]. Employing an external magnetic field, these coated microbial cells gather at specific reactor wall sites, separate from the surrounding solution, and can be reused for treating the same substrate. This approach not only enhances microbial activity and strength but also facilitates their recyclability and recovery, maintenance them against adverse environmental conditions [123]. This process presents a comprehensive strategy, ensuring prolonged and effective biodegradation while enhancing cellular resilience to harsh environmental challenges [124]. This strategy influences the immobilization of microbial cells through NPs, presenting the remarkable synergy between nanotechnology and bioremediation for sustainable developmental solutions.

**Fig. 8:**
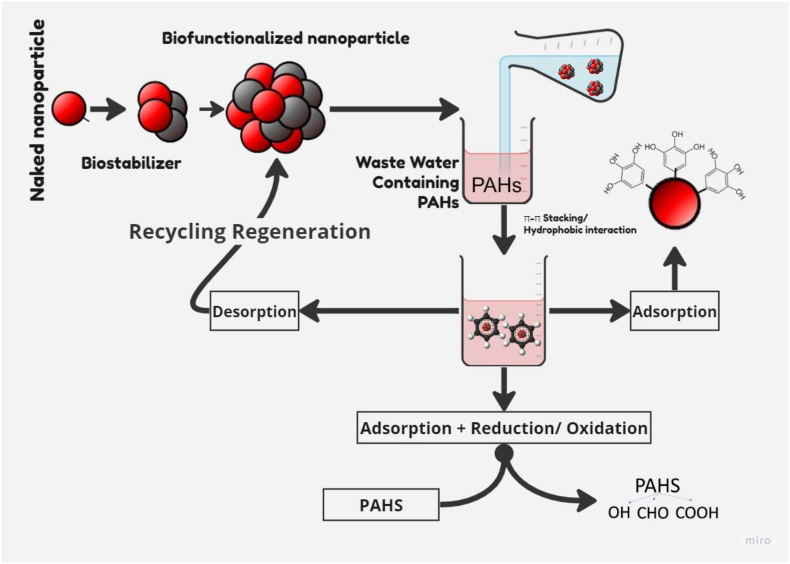
Mechanisms of biofunctionalized nanoparticles in enhancing PAHs degradation.

Biofunctionalized NPs are created by incorporating microbes, enzymes, or biostabilizers onto their surfaces, enhancing their affinity for PAHs. The process entails the initial adsorption of PAHs onto the NPs surface, followed by π–π interactions that drive degradation. After successful degradation, these NPs can be efficiently recycled for additional rounds of PAHs remediation, illustrated in Fig. 8. An exogenous bacterial consortium (*Enterobacter cloacae* and *P*. *otitidis*) immobilized with Fe_3_O_4_ NPs achieved prominent oil degradation efficiencies, with the highest removal rates of total petroleum hydrocarbons, and grease recorded at 85 %, and 83.9 %, respectively, within just 4 h [113].

### 6.2 Microbial enzyme-immobilized NPs

One potential mechanism involves the secretion of specialized enzymes by bacteria, which become immobilized on NPs, facilitating the adsorption of PAHs and subsequently mediating their degradation. Alternatively, bacteria may efficiently degrade PAHs extracellularly, but its efficiency can be upregulated by applying NPs. The nature and strength of interactions between microorganisms and NPs are likely crucial factors influencing the enhanced biodegradation of PAHs [44]. Enzymes functioning as biocatalysts in bioremediation are highly specific and effective, but their practical utility is hindered by instability and short lifetimes [125]. Oxidation reduces enzyme stability and efficiency, limiting their application as cost-effective alternatives to synthetic catalysts. NPs offer a solution to address this by magnifying enzyme stability, longevity, and reusability. Binding enzymes to these magnetic NPs enable easy separation using a magnetic field, significantly prolonging their activity from hours to weeks [126,127]. The study employs trypsin, peroxides, and catabolic enzymes uniformly coated onto core-shell magnetic NPs, demonstrating a remarkable increase in enzyme lifetime and activity. These nanoparticle-enzyme conjugates prove to be more stable, efficient, and cost-effective, shielding enzymes from oxidation. The magnetization of these NPs allows efficient magnetic separation, further enhancing enzyme productivity [128,129].

In a recent study, Deng et al. [130] investigated the potential of immobilized laccase from *T*. *versicolor* for efficient degradation of PAHs. When immobilized on Fe_3_O_4_–SiO_2_-chitosan, the laccase demonstrates distinguished operational stability and reusability, with a capacity of 158 mg/g. Within 48 h, remarkable degradation efficiencies of 81.9 % for ANT and 69.2 % for BAP are achieved. Quantum calculations and mass spectrum analyses reveal anthraquinone and BAP-dione as degradation products. This recoverable magnetic immobilized laccase indicates significant promise for PAH remediation, emphasizing its potential application in environmental restoration.

### 6.3 Integrated nano-biodegradation

Bioremediation has limitations due to its time-dependence, high cost, and low bioavailability, especially in highly contaminated environments with HMW-PAHs. To overcome these challenges, integrated approaches like physical-chemical or physical-biological methods may be employed, combining techniques such as solvent extraction, chemical oxidation, and bioremediation for more effective remediation [131]. Recent advancements in nanotechnology have significantly enhanced integrated remediation approaches by leveraging nano-sized materials to modify the physicochemical properties of contaminants. This technology synergizes with chemical methods like surfactant addition, to increase bioavailability and biological methods, such as biodegradation. However, cautious attention is essential, including choosing non-toxic nanomaterial biomolecules for functionalization and avoiding toxic reducing agents in nanomaterial synthesis. To address this, adopting green and biologically synthesized NPs emerges as a promising solution to mitigate environmental impact [132]. However, biosurfactant-producing bacteria such as *B. subtilis* and iron NPs address the proficient degradation of hazardous PAHs. Biosurfactant enhances PAH bioavailability, aiding bacterial settlement, while iron NPs promote biomass growth and PAH adsorption. This integrated approach achieved an 85 % degradation efficiency for mixed PAHs (ANT, PYR, and BAP) in versatile ecosystems. This strategy highlights the efficient and comprehensive PAH pollutant removal [56].

### 6.4 NPs enhanced microbial activity

NPs have demonstrated the ability to boost microbial activity during the biodegradation of PAHs by creating a conducive microenvironment for microorganisms. Notably, magnetic iron NPs have been observed to enhance cell adhesion, increase nutrient availability, and facilitate metabolic processes, thereby significantly expediting PAH degradation [41,56,133]. Bioaugmentation with graphene oxide-immobilized bacterial pellets (GOBP) enhances PAHs degradation in contaminated soil. High-efficiency degrading bacteria *Paracoccus aminovorans* embedded in GO-alginate-Luria-Bertani composites show 18.51 % higher removal of PAHs (62.86 % over 35 days) than traditional pellets. GOBP focuses on high-molecular-weight PAHs while increasing the abundance of embedded bacteria and enriching potential indigenous degraders like *Pseudarthrobacter* and *Arthrobacter*. This innovative approach offers an advanced technique for remediating organic pollutants in challenging soil environments using bioaugmentation [65]. GO promotes microbial degradation of PAHs, stimulating bacterial growth and gene expression participating in microbial mobility (flagellar assembly), microbial chemotaxis, the two-component system, and phosphotransferase system in soil. In short-term exposure, GO enhances the abundance of degrading microbes, accelerating PAH breakdown. However, extended exposure may lead to degradation saturation. This study indicates GO's impact on microbial PAHs degradation, providing insights into effective environmental remediation and emphasizing the importance of microbial movement and related genetic processes [108].

### 6.5 Nanoparticle-mediated electron transfer

Nano-bioremediation of PAHs employs NPs and zero-valent iron (nZVI) to enhance electron transfer, a crucial step in the degradation process [134]. Electron transfer intensifies the transformation of recalcitrant PAHs into simple and less hazardous intermediates through breaking down complex molecules into simpler. Microbial fuel cells (MFCs) offer a sustainable approach to electricity generation and organic contaminant removal. Carbon nanomaterials, including graphene, reduced graphene oxide, and CNTs, enhance MFC performance due to their increased surface area, conductivity, and electrochemical capacity [134,135]. In a sediment MFC study, reduced graphene oxide -modified anodes exhibited the highest voltage output (30.60–48.61 mV) and PHE removal rates up to 71.2 %. PHE degradation correlated positively with *Pseudomonas, Thauera, Diaphorobacter, Tumebacillus, and Lysobacter* abundances, while PYR degradation correlated with loss on ignition (LOI) degradation. Carbon nanomaterial-modified MFCs show the ability for efficient electricity generation and organic pollutant removal [136]. Lv et al. [137] found enhanced anaerobic-aerobic treatment with nZVI supplemented with rhamnolipid (biosurfactant) and anthraquinone-2,6-disulfonic acid (AQDS) achieved significant degradation rates of 72.81 % for total PAHs and 79.47 % for HMW-PAHs. Key PAHs-degrading bacteria, including *Clostridium*, *Geobacter*, and *Rhodococcus* were dominant contributors. The breakdown route of PAHs, which involves both aerobic and anaerobic processes, was identified by the study of metabolic enzyme function, where nZVI oxidation under anaerobic conditions released effective electron donors for microbial degradation and nZVI-microorganism interactions aided soil pollutant removal. Utilized as an electron shuttle, AQDS facilitates extracellular electron transfer, expediting the exchange between anaerobic bacteria and nZVI. This process enhances system reduction and boosts the microbial transformation rate of soil PAHs [138]. While nZVI is a player, other NPs like Nano-MoO_2_ also activate peroxymonosulfate for PAH derivative degradation [139]. Continuous research aims to refine nano-bioremediation strategies, exploring novel techniques and NPs to achieve more effective PAH degradation.

## 7 Biogenic nanomaterial-mediated PAHs degradation

**Fig. 9:**
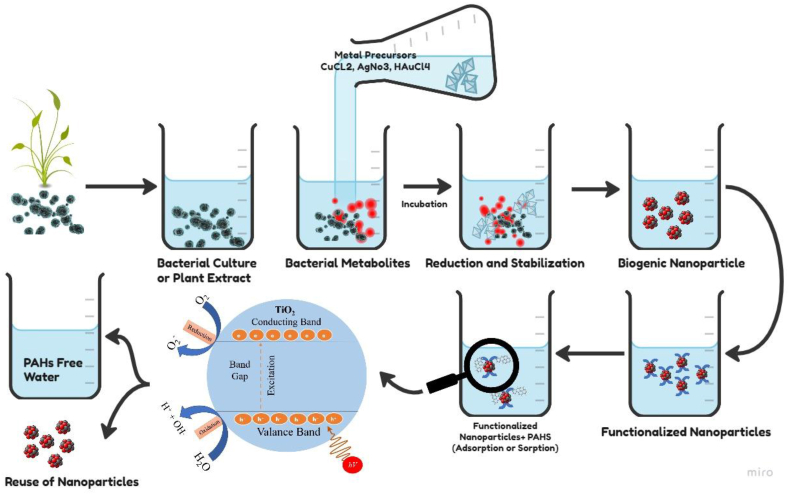
Eco-sustainable degradation of PAHs using biogenic nanomaterials: Synthesis and mechanism.

Biogenic nanomaterials, such as those synthesized using microorganisms and plant extracts, have gained significant attention in the field of green nanotechnology for the remediation of PAHs. These environmentally friendly nanomaterials offer a promising alternative to outdated chemical techniques. In contrast to the time-consuming and challenging top-down approaches, biogenic NPs are bottom-up products formed through biological reduction processes. Microbial enzymes and plant phytochemicals act as efficient reducing agents, facilitating the degradation of PAH contaminants in various environmental matrices. This approach aligns with the principles of green chemistry, emphasizing safety and sustainability [[140], [141], [142]]. Bacterial biogenic nanomaterials have emerged as a potent and eco-green solution for addressing the degradation of PAHs. These nanomaterials are synthesized using various bacterial strains and metabolites, making them environmentally sustainable reducing agents. Biogenic NPs are formed through the addition of a metal precursor to bacterial or plant metabolites, followed by the introduction of ligands or biostabilizers to functionalize the NPs. These functionalized NPs adsorb photons, initiating the degradation of PAHs into harmless metabolites, constituting an environmentally friendly degradation process (Fig. 9). For instance, *A*. *extremophiles* have been employed for the synthesis of crystalline zirconium dioxide (ZrO_2_), where metabolites discharged into the growth medium efficiently reduced ZrOCl_2_ to ZrO_2_ [143]. *Lactobacillus* bacterial strains have been utilized to synthesize titanium dioxide (TiO_2_) NPs [144], while a thermophilic bacterium, *Geobacillus stearothermophilus*, played a pivotal role in the biogenic synthesis of silver (Ag) and gold (Au) NPs [145]. These bacterial strains and their metabolites have been suitable for reducing metal compounds into NPs, facilitating the green remediation of PAH contaminants [146,147]. *B*. *subtilis*, found in rhizosphere soil, exhibited the ability to biosynthesize iron oxide NPs [148]. Moreover, various other bacterial species have demonstrated their capacity to serve as biofactories for synthesizing diverse NPs, including gold, silver, copper, iron, and more.

Fungi have emerged as eco-friendly and economically viable candidates for NPs synthesis due to their unique attributes [149,150]. Ganesan et al. [151] utilized the endophytic fungus *Periconium* sp. for ZnO NPs synthesis, while Clarance et al. [152] employed *Fusarium solani* for gold NPs production, with key roles played by secreted polypeptides and proteins. Kobashigawa et al. (2019) [153] demonstrated the bio-reduction of AgNPs using the ligninolytic fungus *Trametes trogii*, while Gudikandula et al. [154] employed white-rot fungi for AgNP synthesis. *A*. *oryzae* facilitated SeNP reduction from fermented lupin extract [155]. Vago et al. [156] harnessed *Aspergillus, Penicillium, and Trichoderma* fungi for AuNP reduction. Fungi offer high bioaccumulation capacity, metal resistance, ease of handling, and enzymatic capabilities, making them promising for NPs production. Chakravarty et al. [43] investigated the biodegradation potential of ANT was investigated using a novel approach involving green-synthesized TiO_2_ NPs derived from *Paenibacillus* sp. and *Cyperus brevifolius*, known for their PAHs remediation abilities, in conjunction with the bacterium *A*. *faecalis*, isolated from crude oil-contaminated soil. The combined application of TiO_2_ NPs and *A*. *faecalis* led to a considerable reduction in ANT concentration, achieving a 21.3 % decrease in liquid culture after 7 days and a remarkable 37.9 % reduction in soil microcosms over 30 days. GC-MS analysis identified five metabolites, including 1,2-anthracenedihydrodiol, 6,7-benzocoumarin, 3-hydroxy-2-naphthoic acid, salicylic acid, and 9,10-anthraquinone, elucidating a novel ANT biodegradation pathway. These examples illustrate the potential of bacterial biogenic nanomaterials in the sustainable remediation of PAH pollutants, offering a promising avenue for eco-sustainable approaches to tackle this environmental challenge.

## 8 Discussion

This review highlights the role of nanomaterials in bioremediation to remediate PAHs in both liquid and soil samples. It compiles recent research leading to the interpretation of significant advancements in applying both nanomaterials and bioagents for PAH remediation. Bibliographical analysis indicates a growing trend in this research area, though limited studies exist. This review also spotlights the potential and need for further exploration in this field. Elumalai et al. [157] reported the degradation of crude oil by 97 % by the combined effect of iron oxide NPs synthesized using *Aerva lanata* floral part and biosurfactant produced by *Bacillus subtilis* and *Paenibacillus dendritiformis*. Similarly, Muthukumar et al. [158] documented the bacterial species *Pseudomonas aeruginosa* and iron NPs having 5–50 nm size able to synergistically remediate 67 % of ANT. Nickel oxide NPs achieved 79 % degradation of PYR at 2 μg/mL ANT within 60 min under UV and sunlight, confirmed by XRD and SEM analysis showing cubic crystalline structures sized 37–126 nm [159]. Nanomaterials are crucial for addressing hazardous contaminants [125]. Bioelectrochemical systems are versatile, and used in bioremediation, biosensors, microbial fuel cells, and microbial electrolysis cells. When combined with nanotechnology, they significantly enhance the degradation of PAHs. This integration boosts efficiency and effectiveness in environmental cleanup [160]. Besides this advanced research, emphasis is placed on understanding microbial influences on nanomaterials and their effects on toxicity, transport, fate, and bioaccumulation. Developing systems to monitor, detect, and treat trace contaminants in air, water, and soil is crucial for sustainable pollution management [161].

### 8.1 Challenges and consideration

Nanomaterials offer significant advantages in the bioremediation of PAHs due to their high surface area-to-mass ratio, enhanced reactivity, and improved mobility, which enable efficient pollutant degradation. These materials stand out due to their unique characteristics, particularly a significantly increased surface-to-volume ratio as well as enhanced magnetic and catalytic traits compared to their bulk counterparts. Quantum size effects at the nanoscale enhance the efficacy of catalytic processes by modifying electronic structures. They can act as catalysts and be tailored for specific contaminants, making them versatile and cost-effective for large-scale applications. However, potential toxicity to microorganisms, environmental persistence, and high production costs poses challenges. Additionally, the interactions between nanomaterials and microbial cells are not fully understood, and there are regulatory hurdles and risks of unintentional ecological impacts [41,162].

A significant limitation of nanomaterials lies in their potential environmental impact, as their uncontrolled release can lead to widespread harm to abiotic and biotic components of ecosystems, including microorganisms, algae, plants, and animals [163]. Compared to their bulk counterparts, the raised surface area to volume ratio of NPs results in increased reactivity and efficacy; however, their attributes are parallel to their parent chemical species. Consequently, their active participation in diverse physicochemical and biochemical processes within the environment can have detrimental effects on ecological systems. Certain metal NPs such as ZnO, AgO, CuO, and Fe_2_O_3_ are recognized for their toxicity and antimicrobial properties, particularly when present in excessive quantities [164,165]. Challenges include the long-term presence of NPs in the environment their accumulation in organisms, potential for toxicity. The nano-size attributes provide a high surface area and enhanced adsorption capabilities. However, NPs smaller than 20 nm may pose nanotoxicity risks. Small size NPs able to penetrate beneath the microbial cell causing cell malfunction [113].

The challenges also lie in the intricacies of soil complexity. Understanding the behavior of NPs within the soil is a complex task, primarily because of the solid phase nature of the soil and the interactions NPs engage in with soil constituents, including charged humic acids and clay particles. Many studies about NPs have focused on soil suspensions rather than intact soil, as analyzing and characterizing NPs within the soil matrix presents significant difficulties. The utilization of physico-chemical methods to eliminate these NPs from soil and water may prove impractical due to cost inefficiency and environmental concerns. Moreover, the high production costs associated with NPs and their integration with microbial processes pose significant obstacles to scaling up and implementing these techniques on a larger, more practical scale. Hence, a pressing requirement exists to pioneer an eco-friendly, sustainable, and cost-effective bioremediation technology that can specifically target and address these issues. As nano-bioremediation is still in its evolving stage, there is a critical need for standard guidelines governing the application of nanomaterials in bioremediation practices.

### 8.2 Ethical and environmental implications

Health concerns arise from the potential adverse effects of NPs when inhaled, ingested, or absorbed through the skin, raising questions about occupational safety and consumer exposure. The long-term effects of NPs exposure on human health and the environment remain uncertain, requiring ongoing research [166]. It poses significant threats to biological models, and biomarkers including cell death, oxidative stress, DNA damage, apoptosis, and inflammatory responses [167]. Nanomaterial toxicity, which can vary based on size, shape, and surface chemistry, complicates safety assessments. The risk of unintended NPs release during production, use, and disposal poses potential threats to ecosystems and human health. Regulatory and ethical challenges include establishing safety standards, monitoring environmental release, and addressing ethical concerns related to misuse, such as in surveillance and minimizing direct contact with humans. Balancing innovation with ethical considerations and developing strong governance frameworks are essential to ensure the responsible use of nanotechnology in bioremediation [166].

### 8.3 Future research directions

The future of nanomaterials in bioremediation appears promising, as ongoing research is focused on improving their sustainability and efficacy. Improvements in the synthesis and modification of nanomaterials are anticipated to enhance their stability, reactivity, and adsorption capacities in a variety of environmental conditions. The objective of upcoming research is to create nanomaterials that are cost-effective, biodegradable, and non-toxic by optimizing the formulation process at the nanoscale. Furthermore, the integration of nanomaterials with biochar, immobilized enzymes, and electrokinetic methods could provide a variety of approaches to expedite the degradation of PAHs and other contaminants. The potential for eco-friendly and sustainable remediation solutions is further enhanced by the use of biogenic nanomaterials and genetically modified organisms. Understanding the behavior of NPs within soil is a complex task, primarily due to the solid-phase nature of the soil. However, large-scale production of NPs may reduce expenses, and their reusability can make them a realistically cost-effective solution for environmental remediation.

Bioremediation efficacy and microbial metabolic activity can be improved by incorporating “omic” approaches, including transcriptomics, proteomics, and metabolomics. By facilitating the precise monitoring and optimization of remediation processes, the integration of artificial intelligence and machine learning techniques in phytoremediation has the potential to transform the field. The successful implementation of nanomaterials in large-scale bioremediation initiatives will be contingent upon the resolution of challenges related to nanotoxicity, environmental persistence, and implications. In general, the ongoing investigation and innovation in this field hold significant potential for the effective and sustainable management of environmental contamination.

## 9 Conclusion

PAHs, known for their mutagenic and carcinogenic properties, pose substantial threats to human health and ecosystems. Their hazardous nature emphasizes the need for effective remediation methods. Nanomaterials, including metal oxides, CNTs, biopolymers, and nanoscale zeolites, have gained attention due to their potential for remediation. Their small size and significant surface area-to-volume ratio make them valuable tools for improving the adsorption and biodegradation of PAHs in contaminated soil. It is understood from the review that nano-bioremediation exhibits a higher removal efficiency of PAHs compared to traditional bioremediation. This would mean that the application of NPs could enhance the bioremediation process, leading to a more significant reduction in PAH contamination. The strategies such as nanomaterial-assisted microbial degradation, microbial cell immobilization on nanomaterials, and promoting microbial activity through enzymes and electron transfer mediated by nanomaterials provides for enhanced removal of PAHs. These approaches exhibit the versatile role of nanomaterials in upregulating PAH elimination processes, thereby ameliorating the effectiveness of bioremediation techniques.

The use of biogenic nanomaterials, synthesized through microorganisms and plant extracts, offers a sustainable approach to PAH degradation by utilizing microbial enzymes and plant compounds as reducing agents, aligning with green chemistry principles. Future research in nano-bioremediation will likely focus on developing optimization systems, standard protocols, and health safeguard standards for remediating PAHs from the environment. Additionally, standardizing the recovery process of NPs will help reduce production costs. Extending the application of nanomaterials to large-scale bioremediation by integrating other remediation approaches will aid in overcoming challenges and enhancing degradation capability. A standard protocols and regulations might help for a responsible application of nanomaterials in bioremediation endeavors to eliminate PAHs from the environment with a sustainable approach.

## CRediT authorship contribution statement

**Nitu Gupta:** Writing – original draft, Visualization, Software, Resources, Methodology, Investigation, Formal analysis, Data curation, Conceptualization. **Apurba Koley:** Writing – review & editing, Software, Formal analysis, Data curation. **Sandipan Banerjee:** Writing – review & editing, Software, Methodology, Formal analysis, Data curation. **Anudeb Ghosh:** Writing – review & editing, Methodology, Formal analysis, Data curation. **Raza Rafiqul Hoque:** Writing – review & editing, Validation, Supervision, Project administration, Methodology, Conceptualization. **Srinivasan Balachandran:** Writing – review & editing, Validation, Supervision, Project administration, Methodology, Conceptualization.

## Disclosure statements

The authors declare that there are no conflicts of interest as far as they aware of.

## Funding

This work was supported by the 10.13039/501100001407Department of Biotechnology, India, under the 10.13039/501100001407DBT Twinning Project [No. BT/PR25738/NER/95/1329/2017 dated December 24, 2018]. Nitu Gupta acknowledged the funding provided by the 10.13039/501100001501UGC NET-10.13039/100003289JRF Fellowship through 10.13039/501100012624Tezpur University, Tezpur, India. Apurba Koley is financially supported by BEFWAM project: Bioenergy, Fertilizer and Clean water from Invasive Aquatic macrophytes [Grant Ref: BB/S011439/1].

## Ethical Approval

Research does not report on or involve the use of any animal or human data or tissue.

## Consent to participate

Not applicable.

## Consent for publication

All the authors gave consent for the publication of this journal.

## Data availability

Data supporting this study are included within the article.

## Declaration of competing interest

The authors declare that they have no known competing financial interests or personal relationships that could have appeared to influence the work reported in this paper.

## References

[1] Banerjee S., Gupta N., Pramanik K., Gope M., GhoshThakur R., Karmakar A. (2024). Microbes and microbial strategies in carcinogenic polycyclic aromatic hydrocarbons remediation: a systematic review. Environ. Sci. Pollut. Res..

[2] Hussain K., Hoque R.R., Balachandran S., Medhi S., Idris M.G., Rahman M., Hussain F.L. (2018). Monitoring and risk analysis of PAHs in the environment, Handbook. Environ Mater Manage.

[3] Rozaini M.N.H., Khoo K.S., Abdah M.A.A.M., Ethiraj B., Alam M.M., Anwar A.F., Tong W.Y. (2024). Potential application of 2D nano-layered MXene in analysing and remediating endocrine disruptor compounds and heavy metals in water. Environ. Geochem. Health.

[4] Styszko K., Pamuła J., Pac A., Sochacka-Tatara E. (2023). Biomarkers for polycyclic aromatic hydrocarbons in human excreta: recent advances in analytical techniques—a review. Environ. Geochem. Health.

[5] Rouhani A., Gusiatin M.Z., Hejcman M. (2023). An overview of the impacts of coal mining and processing on soil: assessment, monitoring, and challenges in the Czech Republic. Environ. Geochem. Health.

[6] Show B.K., Banerjee S., Banerjee A., GhoshThakur R., Hazra A.K., Mandal N.C., Chaudhury S. (2022). Insect gut bacteria: a promising tool for enhanced biogas production. Rev. Environ. Sci. Biotechnol..

[7] Kuppusamy S., Thavamani P., Venkateswarlu K., Lee Y.B., Naidu R., Megharaj M. (2017). Remediation approaches for polycyclic aromatic hydrocarbons (PAHs) contaminated soils: technological constraints, emerging trends and future directions. Chemosphere.

[8] Pramanik K., Kundu S., Banerjee S., Ghosh P.K., Maiti T.K. (2018). Computational-based structural, functional and phylogenetic analysis of Enterobacter phytases. 3 Biotech.

[9] Zhang Y., Tao S. (2009). Global atmospheric emission inventory of polycyclic aromatic hydrocarbons (PAHs) for 2004. Atmos. Environ..

[10] Ofori S.A., Cobbina S.J., Doke D.A. (2020). The occurrence and levels of polycyclic aromatic hydrocarbons (PAHs) in African environments—a systematic review. Environ. Sci. Pollut. Res..

[11] Hussain K., Hoque R.R. (2015). Seasonal attributes of urban soil PAHs of the Brahmaputra Valley. Chemosphere.

[12] Deka J., Baul N., Bharali P., Sarma K.P., Hoque R.R. (2020). Soil PAHs against varied land use of a small city (Tezpur) of middle Brahmaputra Valley: seasonality, sources, and long-range transport. Environ. Monit. Assess..

[13] Hussain K., Rahman M., Prakash A., Sarma K.P., Hoque R.R. (2016). Atmospheric bulk deposition of PAHs over Brahmaputra Valley: characteristics and influence of meteorology. Aerosol Air Qual. Res..

[14] Deka J., Sarma K.P., Gupta N., Ahmed M.S., Mazumder M.J., Hoque R.R. (2023). Polycyclic aromatic hydrocarbons in groundwater of oil-rich regions of upper Brahmaputra Valley, India: linkages of colloidal transport. Arabian J. Geosci..

[15] Hussain K., Balachandran S., Hoque R.R. (2015). Sources of polycyclic aromatic hydrocarbons in sediments of the bharalu river, a tributary of the river brahmaputra in guwahati, India. Ecotoxicol. Environ. Saf..

[16] Abdel-Shafy H.I., Mansour M.S. (2016). A review on polycyclic aromatic hydrocarbons: source, environmental impact, effect on human health and remediation. Egypt J Petrol.

[17] Hoffman Mills G.L., Latimer J.S., Quinn J.G. (1984). Urban runoff as a source of polycyclic aromatic hydrocarbons to coastal waters. Environ. Sci. Technol..

[18] Skupinska K., Misiewicz I., Kasprzycka-Guttman T. (2004). Polycyclic aromatic hydrocarbons: physicochemical properties, environmental appearance and impact on living organisms. Acta Pol. Pharm..

[19] Wilson L.B., Moran I.L., Anderson K.A., Tanguay R.L. (2023). Advances in PAH mixture toxicology enabled by zebrafish. Curr. Opin. Toxicol..

[20] Lafortune I., Juteau P., Déziel E., Lépine F., Beaudet R., Villemur R. (2009). Bacterial diversity of a consortium degrading high-molecular-weight polycyclic aromatic hydrocarbons in a two-liquid phase biosystem. Microb. Ecol..

[21] Kim K.H., Jahan S.A., Kabir E., Brown R.J. (2013). A review of airborne polycyclic aromatic hydrocarbons (PAHs) and their human health effects. Environ. Int..

[22] Srogi K. (2007). Monitoring of environmental exposure to polycyclic aromatic hydrocarbons: a review. Environ. Chem. Lett..

[23] Zhu Z., Chen Z., Sakurai T., Chiba H., Hui S.P. (2023). Adverse effects of chrysene on human hepatocytes via inducement of oxidative stress and dysregulation of xenobiotic metabolism. Polycycl. Aromat. Comp..

[24] Guo H., Zhang Z., Wang H., Ma H., Hu F., Zhang W. (2021). Oxidative stress and inflammatory effects in human lung epithelial A549 cells induced by phenanthrene, fluorene, and their binary mixture. Environ. Toxicol..

[25] Oyekunle J.A., Inalegwu S.A., Fagbuyi A.O., Adekunle A.S., Ore O.T. (2023). Evaluation of polycyclic aromatic hydrocarbons and potentially toxic metals in commonly consumed pasta products available in the Nigerian Markets. J. Trace Elem. Miner..

[26] Wang Q., Xu X., Cong X., Zeng Z., Xu L., Huo X. (2019). Interactions between polycyclic aromatic hydrocarbons and epoxide hydrolase 1 play roles in asthma. Environ. Geochem. Health.

[27] Samanta S.K., Singh O.V., Jain R.K. (2002). Polycyclic aromatic hydrocarbons: environmental pollution and bioremediation. Trends Biotechnol..

[28] Gupta N., Hoque R.R., Balachandran S. (2024). In bioremediation strategies of chrysene: a carcinogenic polycyclic aromatic hydrocarbons. Rajesh Publication.

[29] Patel A.B., Shaikh S., Jain K.R., Desai C., Madamwar D. (2020). Polycyclic aromatic hydrocarbons: sources, toxicity, and remediation approaches. Front. Microbiol..

[30] Linley S., Thomson N.R. (2021). Environmental applications of nanotechnology: nano-enabled remediation processes in water, soil and air treatment. Water Air Soil Pollut..

[31] Kumari S., Rajput V.D., Sushkova S., Minkina T. (2023). Microbial electrochemical system: an emerging technology for remediation of polycyclic aromatic hydrocarbons from soil and sediments. Environ. Geochem. Health.

[32] Pramanik K., Mandal S., Banerjee S., Ghosh A., Maiti A., Mandal N.C. (2021). Unraveling the heavy metal resistance and biocontrol potential of Pseudomonas sp. K32 strain facilitating rice seedling growth under Cd stress. Chemosphere.

[33] Yousef R., Qiblawey H., El-Naas M.H. (2020). Adsorption as a process for produced water treatment: a review. Processes.

[34] Eldos H.I., Zouari N., Saeed S., Al-Ghouti M.A. (2022). Recent advances in the treatment of PAHs in the environment: application of nanomaterial-based technologies. Arab. J. Chem..

[35] Büyüktiryaki S., Keçili R., Hussain C.M. (2020). Functionalized nanomaterials in dispersive solid phase extraction: advances & prospects. TrAC Trends Anal Chem.

[36] Basak G., Hazra C., Sen R. (2020). Biofunctionalized nanomaterials for in situ clean-up of hydrocarbon contamination: a quantum jump in global bioremediation research. J. Environ. Manag..

[37] Chauhan P., Imam A., Kanaujia P.K., Suman S.K. (2023). Nano-bioremediation: an eco-friendly and effective step towards petroleum hydrocarbon removal from environment. Environ. Res..

[38] Yang G., Jiang Y., Yin B., Liu G., Ma D., Zhang G. (2023). Efficiency and mechanism on photocatalytic degradation of fluoranthene in soil by Z-scheme g-C3N4/α-Fe2O3 photocatalyst under simulated sunlight. Environ. Sci. Pollut. Res..

[39] Sun Y., Wang K., Chen D., Xu Q., Li N., Li H., Lu J. (2023). Activation of persulfate by highly dispersed FeCo bimetallic alloy for in-situ remediation of polycyclic aromatic hydrocarbon-contaminated soil. Sep. Purif. Technol..

[40] Banerjee S., Maiti T.K., Roy R.N. (2022). Enzyme producing insect gut microbes: an unexplored biotechnological aspect. Crit. Rev. Biotechnol..

[41] Rajput V.D., Kumari S., Minkina T., Sushkova S., Mandzhieva S. (2023). Nano-enhanced microbial remediation of PAHs contaminated soil. Air Soil. Water Res..

[42] Mohan D., Sarswat A., Ok Y.S., Pittman C.U. (2014). Organic and inorganic contaminants removal from water with biochar, a renewable, low cost and sustainable adsorbent–a critical review. Bioresour. Technol..

[43] Chakravarty P., Deka H., Chowdhury D. (2023). Anthracene removal potential of green synthesized titanium dioxide nanoparticles (TiO2-NPs) and Alcaligenes faecalis HP8 from contaminated soil. Chemosphere.

[44] Gulia S., Kothari V., Verma S.R., Das A., Singh B.P., Chandra P. (2023). Nanobiotechnology for Bioremediation.

[45] Koley A., Mukhopadhyay P., Gupta N., Singh A., Ghosh A., Show B.K. (2023). Biogas production potential of aquatic weeds as the next-generation feedstock for bioenergy production: a review. Environ. Sci. Pollut. Res..

[46] Gupta N., Banerjee S., Koley A., Basu A., Gogoi N., Hoque R.R. (2024). Bioremediation for Sustainable Environmental Cleanup.

[47] Koley A., Ghosh A., Banerjee S., Gupta N., Thakur R.G., Show B.K. (2024). Bioremediation for Sustainable Environmental Cleanup.

[48] Kegere J., Alblooshi A., Nguyen H.L., Alnaqbi M.A. (2023). Immobilized bacterial cells on electrospun nanofibers for crude oil spills treatment and bioremediation. ChemNanoMat.

[49] Dai Y., Yin L., Niu J. (2011). Laccase-carrying electrospun fibrous membranes for adsorption and degradation of PAHs in shoal soils. Environ. Sci. Technol..

[50] Dai Y., Niu J., Yin L., Xu J., Xu J. (2013). Laccase-carrying electrospun fibrous membrane for the removal of polycyclic aromatic hydrocarbons from contaminated water. Sep. Purif. Technol..

[51] Al-Zaban M.I., Mahmoud M.A., AlHarbi M.A., Bahatheq A.M. (2020). Bioremediation of crude oil by rhizosphere fungal isolates in the presence of silver nanoparticles. Int. J. Environ. Res. Publ. Health.

[52] Gupta H., Gupta B. (2015). Photocatalytic degradation of polycyclic aromatic hydrocarbon benzo[a]pyrene by iron oxides and identification of degradation products. Chemosphere.

[53] McQueen A.D., Ballentine M.L., May L.R., Laber C.H., Das A., Bortner M.J., Kennedy A.J. (2021). Photocatalytic degradation of polycyclic aromatic hydrocarbons in water by 3D printed TiO2 composites. ACS ES&T Water.

[54] El-Sheshtawy H.S., Ahmed W. (2017). Bioremediation of crude oil by Bacillus licheniformis in the presence of different concentration nanoparticles and produced biosurfactant. Int. J. Environ. Sci. Technol..

[55] Oyewole O.A., Raji R.O., Musa I.O., Enemanna C.E., Abdulsalam O.N., Yakubu J.G. (2019).

[56] Parthipan P., Cheng L., Dhandapani P., Elumalai P., Huang M., Rajasekar A. (2022). Impact of biosurfactant and iron nanoparticles on biodegradation of polyaromatic hydrocarbons (PAHs). Environ. Pollut..

[57] Alothman Z.A., Wabaidur S.M. (2019). Application of carbon nanotubes in extraction and chromatographic analysis: a review. Arab. J. Chem..

[58] Zhang J., Yu F., Ke X., Yu H., Guo P., Du L. (2022). Carbon quantum dots bridged TiO2/CdIn2S4 toward photocatalytic upgrading of polycyclic aromatic hydrocarbons to benzaldehyde. Molecules.

[59] Devi P., Saini S., Kim K.H. (2019). The advanced role of carbon quantum dots in nanomedical applications. Biosens. Bioelectron..

[60] Carrillo-Carrión C., Simonet B.M., Valcárcel M. (2009). Carbon nanotube–quantum dot nanocomposites as new fluorescence nanoparticles for the determination of trace levels of PAHs in water. Anal. Chim. Acta.

[61] Firoozbakht M., Sepahi A.A., Rashedi H., Yazdian F. (2022). Investigating the effect of nanoparticle on phenanthrene biodegradation by Labedella gwakjiensis strain KDI. Biodegradation.

[62] Zhao J., Wang Z., Zhao Q., Xing B. (2014). Adsorption of phenanthrene on multilayer graphene as affected by surfactant and exfoliation. Environ. Sci. Technol..

[63] Mahpishanian S., Sereshti H., Ahmadvand M. (2017). A nanocomposite consisting of silica-coated magnetite and phenyl-functionalized graphene oxide for extraction of polycyclic aromatic hydrocarbon from aqueous matrices. J. Environ. Sci..

[64] Wu G., Liu X., Zhou P., Wang L., Hegazy M., Huang X., Huang Y. (2019). A facile approach for the reduction of 4 nitrophenol and degradation of Congo red using gold nanoparticles or laccase decorated hybrid inorganic nanoparticles/polymer-biomacromolecules vesicles. Mater. Sci. Eng. C.

[65] Ren W., Liu H., Mao T., Teng Y., Zhao R., Luo Y. (2022). Enhanced remediation of PAHs-contaminated site soil by bioaugmentation with graphene oxide immobilized bacterial pellets. J. Hazard Mater..

[66] Dutta V., Devasia J., Chauhan A., Jayalakshmi M., Vasantha V., Jha A., Ghotekar S. (2022). Photocatalytic nanomaterials: applications for remediation of toxic polycyclic aromatic hydrocarbons and green management. Chem Eng J Adv.

[67] Sharma A., Siddiqi Z.M., Pathania D. (2017). Adsorption of polyaromatic pollutants from water system using carbon/ZnFe2O4 nanocomposite: equilibrium, kinetic and thermodynamic mechanism. J. Mol. Liq..

[68] Laurent S., Forge D., Port M., Roch A., Robic C., Vander Elst L., Muller R.N. (2008). Magnetic iron oxide nanoparticles: synthesis, stabilization, vectorization, physicochemical characterizations, and biological applications. Chem. Rev..

[69] Al-Hunaiti A., Ghazzy A.M., Mahmoud N.T. (2024). Photocatalytic Polyaromatic hydrocarbons (PAH) utilizing magnetic CrFe2O4 nanoparticle: green synthesis, characterization, ab initio studies, electronic, magnetic features and water treatment application. Chem. Eng. J. Adv..

[70] Inbaraj B.S., Sridhar K., Chen B.H. (2021). Removal of polycyclic aromatic hydrocarbons from water by magnetic activated carbon nanocomposite from green tea waste. J. Hazard Mater..

[71] Mukwevho N., Gusain R., Fosso-Kankeu E., Kumar N., Waanders F., Ray S.S. (2020). Removal of naphthalene from simulated wastewater through adsorption-photodegradation by ZnO/Ag/GO nanocomposite. J. Ind. Eng. Chem..

[72] Rani M., Shanker U. (2020). Metal oxide-chitosan based nanocomposites for efficient degradation of carcinogenic PAHs. J. Environ. Chem. Eng..

[73] Rani M., Shanker U. (2019). Mineralization of carcinogenic anthracene and phenanthrene by sunlight active bimetallic oxides nanocomposites. J. Colloid Interface Sci..

[74] Osadebe A.U., Ogugbue C.J., Okpokwasili G.C. (2024). Bioremediation of crude oil polluted surface water using specialised alginate-based nanocomposite beads loaded with hydrocarbon-degrading bacteria and inorganic nutrients. Ann. Finance.

[75] Russo V., Hmoudah M., Broccoli F., Iesce M.R., Jung O.S., Di Serio M. (2020). Applications of metal organic frameworks in wastewater treatment: a review on adsorption and photodegradation. Front. Chem. Eng..

[76] Dhaka S., Kumar R., Deep A., Kurade M.B., Ji S.W., Jeon B.H. (2019). Metal–organic frameworks (MOFs) for the removal of emerging contaminants from aquatic environments. Coord. Chem. Rev..

[77] Li H., Yao Y., Zhang J., Du J., Xu S., Wang C., Zhou J. (2020). Degradation of phenanthrene by peroxymonosulfate activated with bimetallic metal-organic frameworks: kinetics, mechanisms, and degradation products. Chem. Eng. J..

[78] Zhang X., Zhang X., Cai Y., Wang S. (2022). Peroxymonosulfate-activated molecularly imprinted bimetallic MOFs for targeted removal of PAHs and recovery of biosurfactants from soil washing effluents. Chem. Eng. J..

[79] Liu M., Zhang L., Yang R., Cui H., Li Y., Li X., Huang H. (2024). Integrating metal-organic framework ZIF-8 with green modifier empowered bacteria with improved bioremediation. J. Hazard Mater..

[80] Kumari B., Singh D.P. (2016). A review on multifaceted application of nanoparticles in the field of bioremediation of petroleum hydrocarbons. Ecol. Eng..

[81] Aljaerani H.A., Samykano M., Saidur R., Pandey A.K., Kadirgama K. (2021). Nanoparticles as molten salts thermophysical properties enhancer for concentrated solar power: a critical review. J. Energy Storage.

[82] Liu X., Wang D., Li Y. (2012). Synthesis and catalytic properties of bimetallic nanomaterials with various architectures. Nano Today.

[83] Konnova S.A., Lvov Y.M., Fakhrullin R.F. (2016). Nanoshell assembly for magnet-responsive oil-degrading bacteria. Langmuir.

[84] Li H., Jian S., Baalousha M. (2023). Applications of catalytic nanomaterials in energy and environment. Molecules.

[85] Nisha S., Karthick S.A., Gobi N. (2012). A review on methods, application and properties of immobilized enzyme. Chem. Sci. Rev. Lett..

[86] Acevedo F., Pizzul L., González M.E., Cea M., Gianfreda L., Diez M.C. (2010). Degradation of polycyclic aromatic hydrocarbons by free and nanoclay-immobilized manganese peroxidase from Anthracophyllum discolor. Chemosphere.

[87] Shyamalagowri S., Bhavithra H.A., Akila N., Jeyaraj S.S.G., Aravind J., Kamaraj M., Pandiaraj S. (2024). Carbon-based adsorbents for the mitigation of polycyclic aromatic hydrocarbon: a review of recent research. Environ. Geochem. Health.

[88] Reghunadhan A., Kalarikkal N., Thomas S. (2018). Characterization of Nanomaterials.

[89] Heisnam P., Moirangthem A., Singh Y.D., Dutta P., Devi C.V., Hazarika B.N. (2022). Bioremediation.

[90] Yadav K.K., Singh J.K., Gupta N., Kumar V. (2017). A review of nanobioremediation technologies for environmental cleanup: a novel biological approach. J. Mater. Environ. Sci..

[91] González-Ramírez D.F., Ávila-Pérez P., Torres-Bustillos L.G., Aguilar-López R., Esparza-García F.J., Rodríguez-Vázquez R. (2017). Removal of phenanthrene in an aqueous matrix by entrapped crude enzymes on alginate beads combined with TiO2-C-Ag coated fiberglass. Int. J. Environ. Sustain Dev..

[92] Li D., Fang Y., Lu J., Sun J., Zhao X., Hou N., Xing J. (2023). Enhanced biodegradation of PAHs by biochar and a TiO2@ biochar composite under light irradiation: photocatalytic mechanism, toxicity evaluation and ecological response. Chem. Eng. J..

[93] Zhao Z., Chen W., Cheng Y., Li J., Chen Z. (2023). Burkholderia cepacia immobilized onto rGO as a biomaterial for the removal of naphthalene from wastewater. Environ. Res..

[94] Davoodi S.M., Miri S., Brar S.K., Martel R. (2023). Continuous fixed-bed column studies to remove polycyclic aromatic hydrocarbons by degrading enzymes immobilized on polyimide aerogels. J. Water Process Eng..

[95] Zhou H., Li X., Hu B., Wu M., Zhang Y., Yi X., Liu Y. (2021). Assembly of fungal mycelium-carbon nanotube composites and their application in pyrene removal. J. Hazard Mater..

[96] Qin Z., Zhao Z., Jiao W., Han Z., Xia L., Fang Y., Jiang Y. (2020). Phenanthrene removal and response of bacterial community in the combined system of photocatalysis and PAH-degrading microbial consortium in laboratory system. Bioresour. Technol..

[97] Qin Z., Zhao Z., Jiao W., Han Z., Xia L., Fang Y., Jiang Y. (2020). Coupled photocatalytic-bacterial degradation of pyrene: removal enhancement and bacterial community responses. Environ. Res..

[98] Ojha N., Mandal S.K., Das N. (2019). Enhanced degradation of indeno (1, 2, 3-cd) pyrene using Candida tropicalis NN4 in presence of iron nanoparticles and produced biosurfactant: a statistical approach. 3 Biotech.

[99] Mandal S.K., Ojha N., Das N. (2018). Process optimization of benzo [ghi] perylene biodegradation by yeast consortium in presence of ZnO nanoparticles and produced biosurfactant using Box-Behnken design. Front. Biol..

[100] Mandal S.K., Ojha N., Das N. (2018). Optimization of process parameters for the yeast mediated degradation of benzo [a] pyrene in presence of ZnO nanoparticles and produced biosurfactant using 3-level Box-Behnken design. Ecol. Eng..

[101] Tarafdar A., Sarkar T.K., Chakraborty S., Sinha A., Masto R.E. (2018). Biofilm development of Bacillus thuringiensis on MWCNT buckypaper: adsorption-synergic biodegradation of phenanthrene. Ecotoxicol. Environ. Saf..

[102] She A., Tao X., Huang T., Lu G., Zhou Z., Guo C., Dang Z. (2016). Effects of nano bamboo charcoal on PAHs-degrading strain Sphingomonas sp. GY2B, Ecotoxicol. Environ. Saf..

[103] Gan X., Teng Y., Zhao L., Ren W., Chen W., Hao J. (2018). Influencing mechanisms of hematite on benzo(a)pyrene degradation by the PAH-degrading bacterium Paracoccus sp. Strain HPD-2: insight from benzo(a)pyrene bioaccessibility and bacteria activity. J. Hazard Mater..

[104] Tao X.Q., Wang J., Duan X.C., Zou M.Y., Du J., Zhang J.L., Lu G.N. (2021). Interactions between polycyclic aromatic hydrocarbons (PAHs)-degrading strain Sphingomonas sp. GY2B and nano bamboo charcoal. Desalination Water Treat..

[105] Li L., Zhang X., Zhu P., Yong X., Wang Y., An W., Zhou J. (2021). Enhancing biomethane production and pyrene biodegradation by addition of bio-nano FeS or magnetic carbon during sludge anaerobic digestion. Environ. Technol..

[106] Ye Q., Zhang Z., Huang Y., Fang T., Cui Q., He C., Wang H. (2018). Enhancing electron transfer by magnetite during phenanthrene anaerobic methanogenic degradation. Int. Biodeterior. Biodegrad..

[107] Li L., Liu R., Chen J., Tai P., Bi X., Zou P., Xiao Y. (2023). Biotrophic interactions between plant and endophytic bacteria in removal of PAHs and Cd from contaminated soils enhanced by graphene oxide. J. Clean. Prod..

[108] Xue C., Li L., Guo C., Gao Y., Yang C., Deng X., Sun L. (2023). Understanding the role of graphene oxide in affecting PAHs biodegradation by microorganisms: an integrated analysis using 16SrRNA, metatranscriptomic, and metabolomic approaches. J. Hazard Mater..

[109] Cruz Viggi C., Tucci M., Resitano M., Palushi V., Crognale S., Matturro B., Aulenta F. (2023). Enhancing the anaerobic biodegradation of petroleum hydrocarbons in soils with electrically conductive materials. Bioengineering.

[110] Chai C., Ji Y., Wang N., Ge W., Wu J., Wang Y.Q., Li Y. (2023). Immobilized lignin peroxidase on chitosan-modified halloysite nanotubes for degradation of polycyclic aromatic hydrocarbons in soil. Int. J. Environ. Sci. Technol..

[111] Wang A., Li Y., Tan H., Zhang A., Xie Y., Wu B., Xu H. (2019). A novel microbe consortium, nano-visible light photocatalyst and microcapsule system to degrade PAHs. Chem. Eng. J..

[112] Jorfi S., Samaei M.R., Soltani R.D.C., Talaie Khozani A., Ahmadi M., Barzegar G., Mehrabi N. (2017). Enhancement of the bioremediation of pyrene-contaminated soils using a hematite nanoparticle-based modified fenton oxidation in a sequenced approach. Soil Sediment Contam Int J.

[113] Bestawy E.E., El-Shatby B.F., Eltaweil A.S. (2020). Integration between bacterial consortium and magnetite (Fe3O4) nanoparticles for the treatment of oily industrial wastewater. World J. Microbiol. Biotechnol..

[114] Sekoai P.T., Ouma C.N.M., Du Preez S.P., Modisha P., Engelbrecht N., Bessarabov D.G., Ghimire A. (2019). Application of nanoparticles in biofuels: an overview. Fuel.

[115] Wu Y., Pang H., Liu Y., Wang X., Yu S., Fu D. (2019). Environmental remediation of heavy metal ions by novel-nanomaterials: a review. Environ. Pollut..

[116] El-Sayyad G.S., Elfadil D., Mosleh M.A., Hasanien Y.A., Mostafa A., Abdelkader R.S. (2024). Eco-friendly strategies for biological synthesis of green nanoparticles with promising applications. BioNanoScience.

[117] Kapoor R.T., Salvadori M.R., Rafatullah M., Siddiqui M.R., Khan M.A., Alshareef S.A. (2021). Exploration of microbial factories for synthesis of nanoparticles–a sustainable approach for bioremediation of environmental contaminants. Front. Microbiol..

[118] Feng J.R., Deng Q.X., Han S.K., Ni H.G. (2023). Use of nanoparticle-coated bacteria for the bioremediation of organic pollution: a mini review. Chemosphere.

[119] Moghadam M.J., Moayedi H., Sadeghi M.M., Hajiannia A. (2016). A review of combinations of electrokinetic applications. Environ. Geochem. Health.

[120] Ghosal D., Ahn Y. (2016). Current state of knowledge in microbial degradation of polycyclic aromatic hydrocarbons (PAHs): a review. Front. Microbiol..

[121] Bayat Z., Hassanshahian M., Cappello S. (2015). Immobilization of microbes for bioremediation of crude oil polluted environments: a mini review. Open Microbiol. J..

[122] Li Y.G., Gao H.S., Li W.L., Xing J.M., Liu H.Z. (2009). In situ magnetic separation and immobilization of dibenzothiophene-desulfurizing bacteria. Bioresour. Technol..

[123] Shan G., Xing J., Zhang H., Liu H. (2005). Biodesulfurization of dibenzothiophene by microbial cells coated with magnetite nanoparticles. Appl. Environ. Microbiol..

[124] Rizwan M.D., Singh M., Mitra C.K., Morve R.K. (2014). Ecofriendly application of nanomaterials: nanobioremediation. J. Nanopart..

[125] Shahi M.P., Kumari P., Mahobiya D., Shahi S.K. (2021). Nano-bioremediation of environmental contaminants: applications, challenges, and future prospects. Bioremediation for Environmental Sustainability.

[126] Matveeva V.G., Bronstein L.M. (2021). Magnetic nanoparticle-containing supports as carriers of immobilized enzymes: key factors influencing the biocatalyst performance. Nanomaterials.

[127] Biswal T. (2023). Phytoremediation: Management of Environmental Contaminants.

[128] Lau E.C., Yiu H.H. (2022). Nanomaterials for Biocatalysis.

[129] Qiang Y., Sharma A., Paszczynski A., Meyer D. (2007). Proceedings of the 2007 NSTI Nanotechnology Conference and Trade Show.

[130] Deng J., Wang H., Zhan H., Wu C., Huang Y., Yang B., Ling W. (2022). Catalyzed degradation of polycyclic aromatic hydrocarbons by recoverable magnetic chitosan immobilized laccase from Trametes versicolor. Chemosphere.

[131] Kuppusamy S., Palanisami T., Megharaj M., Venkateswarlu K., Naidu R. (2016). In-situ remediation approaches for the management of contaminated sites: a comprehensive overview. Rev. Environ. Contam. Toxicol..

[132] Parthipan P., Prakash C., Perumal D., Elumalai P., Rajasekar A., Cheng L. (2021). Biogenic nanoparticles and strategies of nano-bioremediation to remediate PAHs for a sustainable future. Biotechnol. Sustain. Environ..

[133] Gutierrez A.M., Dziubla T.D., Hilt J.Z. (2017). Recent advances on iron oxide magnetic nanoparticles as sorbents of organic pollutants in water and wastewater treatment. Rev. Environ. Health.

[134] Liu Y., Zhang X., Zhang Q., Li C. (2020). Microbial fuel cells: nanomaterials based on anode and their application. Energy Technol..

[135] Wilberforce T., Abdelkareem M.A., Elsaid K., Olabi A.G., Sayed E.T. (2022). Role of carbon-based nanomaterials in improving the performance of microbial fuel cells. Energy.

[136] Liang Y., Zhai H., Liu B., Ji M., Li J. (2020). Carbon nanomaterial-modified graphite felt as an anode enhanced the power production and polycyclic aromatic hydrocarbon removal in sediment microbial fuel cells. Sci. Total Environ..

[137] Lv L., Sun L., Yuan C., Han Y., Huang Z. (2022). The combined enhancement of RL, nZVI and AQDS on the microbial anaerobic-aerobic degradation of PAHs in soil. Chemosphere.

[138] Liu X., Li Z., Zhang C., Tan X., Yang X., Wan C., Lee D.J. (2020). Enhancement of anaerobic degradation of petroleum hydrocarbons by electron intermediate: performance and mechanism. Bioresour. Technol..

[139] Chen X., Vione D., Borch T., Wang J., Gao Y. (2021). Nano-MoO2 activates peroxymonosulfate for the degradation of PAH derivatives. Water Res..

[140] Owaid M.N. (2019). Green synthesis of silver nanoparticles by Pleurotus (oyster mushroom) and their bioactivity. Environ. Nanotechnol. Monit. Manag..

[141] Majlesi Z., Ramezani M., Gerami M. (2018). Investigation on some main glycosides content of Stevia rebaudian B. under different concentrations of commercial and synthesized silver nanoparticles. Pharm. Biomed. Res..

[142] Iravani S. (2014). Bacteria in nanoparticle synthesis: current status and future prospects. Int. Sch. Res. Notices.

[143] Suriyaraj S.P., Ramadoss G., Chandraraj K., Selvakumar R. (2019). One pot facile green synthesis of crystalline bio-ZrO2 nanoparticles using Acinetobacter sp. KCSI1 under room temperature. Mater. Sci. Eng. C.

[144] Jha A.K., Prasad K. (2010). Biosynthesis of metal and oxide nanoparticles using Lactobacilli from yoghurt and probiotic spore tablets. Biotechnol. J..

[145] A.M. Fayaz, M. Girilal, M. Rahman, R. Venkatesan, P.T. Kalaichelvan, Biosynthesis of silver and gold nanoparticles using thermophilic bacterium Geobacillus stearothermophilus, Process Biochem. 46.

[146] Zhang H., Hu X. (2018). Biosynthesis of Pd and Au as nanoparticles by a marine bacterium Bacillus sp. GP and their enhanced catalytic performance using metal oxides for 4-nitrophenol reduction. Enzym. Microb. Technol..

[147] Srivastava N., Mukhopadhyay M. (2013). Biosynthesis and structu ral characterization of selenium nanoparticles mediated by Zooglea ramigera. Powder Technol..

[148] Sundaram P.A., Augustine R., Kannan M. (2012). Extracellular biosynthesis of iron oxide nanoparticles by Bacillus subtilis strains isolated from rhizosphere soil. Biotechnol. Bioproc. Eng..

[149] Vahabi K., Mansoori G.A., Karimi S. (2011). Biosynthesis of silver nanoparticles by fungus Trichoderma reesei (a route for large-scale production of AgNPs). Insciences J.

[150] Menon S., Rajeshkumar S., Kumar V. (2017). A review on biogenic synthesis of gold nanoparticles, characterization, and its applications. Resour-Efficient Technol..

[151] Ganesan V., Hariram M., Vivekanandhan S., Muthuramkumar S. (2020). Periconium sp. (endophytic fungi) extract mediated sol-gel synthesis of ZnO nanoparticles for antimicrobial and antioxidant applications. Mater. Sci. Semicond. Process..

[152] Clarance P., Luvankar B., Sales J., Khusro A., Agastian P., Tack J.C., Kim H.J. (2020). Green synthesis and characterization of gold nanoparticles using endophytic fungi Fusarium solani and its in-vitro anticancer and biomedical applications. Saudi J. Biol. Sci..

[153] Kobashigawa J.M., Robles C.A., Ricci M.L.M., Carmarán C.C. (2019). Influence of strong bases on the synthesis of silver nanoparticles (AgNPs) using the ligninolytic fungi Trametes trogii. Saudi J. Biol. Sci..

[154] Gudikandula K., Vadapally P., Charya M.S. (2017). Biogenic synthesis of silver nanoparticles from white rot fungi: their characterization and antibacterial studies. Open.

[155] Mosallam F.M., El-Sayyad G.S., Fathy R.M., El-Batal A.I. (2018). Biomolecules-mediated synthesis of selenium nanoparticles using Aspergillus oryzae fermented Lupin extract and gamma radiation for hindering the growth of some multidrug-resistant bacteria and pathogenic fungi. Microb. Pathog..

[156] Vágó A., Szakacs G., Sáfrán G., Horvath R., Pécz B., Lagzi I. (2016). One-step green synthesis of gold nanoparticles by mesophilic filamentous fungi. Chem. Phys. Lett..

[157] Elumalai P., Rajamohan R., Purayil A.T.V., Menon V., Srivatsan R.P., Kumar A.S. (2024). Biosurfactant and iron oxide nanoparticle-assisted bioremediation of soil co-contaminated with hydrocarbons and hazardous heavy metals. Chem. Eng. J..

[158] Muthukumar B., Satheeshkumar A., Parthipan P., Laishram B., Duraimurugan R., Devanesan S. (2024). Integrated approach of nano assisted biodegradation of anthracene by Pseudomonas aeruginosa and iron oxide nanoparticles. Environ. Res..

[159] Brindhadevi K., Kim T.P., Alharbi S.A., Ramesh M.D., Lee J., Bharathi D. (2024). Enhanced photocatalytic degradation of polycyclic aromatic hydrocarbons (PAHs) Using NiO nanoparticles. Environ. Res..

[160] Khan R., Bhadra S., Nayak S., Bindu A., Prabhu A.A., Sevda S. (2024). Emerging Trends in fabrication and modification techniques for bioelectrochemical system electrodes: a review. J. Taiwan Inst. Chem. Eng..

[161] Ningombam L., Mana T., Apum G., Ningthoujam R., Singh Y.D. (2024). Nano-bioremediation: a prospective approach for environmental decontamination in focus to soil, water and heavy metals. Environ. Nanotechnol. Monit. Manag..

[162] Asghar N., Hussain A., Nguyen D.A., Ali S., Hussain I., Junejo A., Ali A. (2024). Advancement in nanomaterials for environmental pollutants remediation: a systematic review on bibliometrics analysis, material types, synthesis pathways, and related mechanisms. J. Nanobiotechnol..

[163] Handy R.D., Von der Kammer F., Lead J.R., Hassellöv M., Owen R., Crane M. (2008). The ecotoxicology and chemistry of manufactured nanoparticles. Ecotoxicology.

[164] Azam A., Ahmed A.S., Oves M., Khan M.S., Habib S.S., Memic A. (2012). Antimicrobial activity of metal oxide nanoparticles against Gram-positive and Gram-negative bacteria: a comparative study. Int. J. Nanomed..

[165] Zhou Y., Kong Y., Kundu S., Cirillo J.D., Liang H. (2012). Antibacterial activities of gold and silver nanoparticles against Escherichia coli and bacillus Calmette-Guérin. J. Nanobiotechnol..

[166] Kumah E.A., Fopa R.D., Harati S., Boadu P., Zohoori F.V., Pak T. (2023). Human and environmental impacts of nanoparticles: a scoping review of the current literature. BMC Publ. Health.

[167] Sahu M.K., Yadav R., Tiwari S.P. (2023). Recent advances in nanotechnology. Int. J. Nanomater. Nanotechnol. Nanomedicine.

