# Supplementary material for: Nanomaterial-mediated strategies for enhancing bioremediation of polycyclic aromatic hydrocarbons: A systematic review
Source: Hybrid Adv. 2024 Dec;7:None. doi: 10.1016/j.hybadv.2024.100315 (PMC11698305; doi:10.1016/j.hybadv.2024.100315)
Supplement: Multimedia component 1 [file mmc1.docx]

**Supplementary Material**

**Nanomaterial-mediated Strategies for boosting Bioremediation of Polycyclic Aromatic Hydrocarbons: A Systematic Review**

*Nitu Gupta^1^, Apurba Koley^2^, Sandipan Banerjee^3^Anudeb Ghosh^2^, Raza Rafiqul Hoque^1*^, and Srinivasan Balachandran^2^**

*^1^*Department of Environmental Science, Tezpur University, Napaam, Tezpur, Assam, 784028, India

*^2^*Department of Environmental Studies, Visva-Bharati, Santiniketan-731235, West Bengal, India

*^3^*Mycology and Plant Pathology Laboratory, Department of Botany, Visva-Bharati, Santiniketan, 731235, West Bengal, India

***Author and Co-authors:***

**Nitu Gupta**

Email: [nituevs@gmail.com](mailto:nituevs@gmail.com)

ORCID ID: 0000-0002-1462-2076

**Apurba Koley**

E-mail: [apurbakoley1@gmail.com](mailto:apurbakoley1@gmail.comn)

ORCID ID: 0000-0002-5785-626X

**Sandipan Banerjee**

Email: [sandipanmicbio@gmail.com](mailto:sandipanmicbio@gmail.com)

ORCID ID: 0000-0002-2102-8809

**Anudeb Ghosh**

Email: [anudev.ghosh@gmail.com](mailto:anudev.ghosh@gmail.com)

ORCID: 0000-0002-6915-1507

****Corresponding author:***

**Dr. Srinivasan Balachandran**

E-mail: [s.balachandran@visva-bharati.ac.in](mailto:s.balachandran@visva-bharati.ac.in)

ORCID ID: 0000-0003-4247-408X

**Prof. Raza Rafiqul Hoque**

E-mail: [rrh@tezu.ernet.in](mailto:rrh@tezu.ernet.in)

ORCID ID: 0000-0002-6375-9431

Table S1: Detailed Comparative account of Bioremediation and Nano-Bioremediation for PAHs degradation in liquid samples

| References | Remediation method | Type of NP | NP Conc. | Single /Mix inoculation | Bioagent | PAHs type | PAHs degradation (%) | Initial conc. (mg L^-1^) | Final Conc. (mg L^-1^) | Temperature (°C) | Duration | pH | Supplement | Self-spiked PAHs/contaminated |
| --- | --- | --- | --- | --- | --- | --- | --- | --- | --- | --- | --- | --- | --- | --- |
| [61] | Bioremediation | - | - | Single | *Labedella gwakjiensis* | PHE | 37.12 | 527.5 | 331.69 | 30 | 72 h | 7–7.2 | Yes | Self-spiked |
|  | Nano-Bioremediation | CQDs.Fe_3_O_4_ | \| 0.5g/L \| \| --- \| | Single | *Labedella gwakjiensis* | PHE | 81.77 | 527.5 | 96.16 | 30 | 72 h | 7–7.2 | Yes | Self-spiked |
| [43] | Bioremediation | - | - | Single | *Alcaligenes faecalis* | ANT | 10.4 | 500 | 448±2 | 35±2 | 7 d | 7 | Yes | Self-spiked |
|  | Nano-Bioremediation | TiO_2_ NPs | 150 mg /L | Single | *Alcaligenes faecalis* | ANT | 21.3 | 500 | 393.5±18 | 35±2 | 7 d | 7 | Yes | Self-spiked |
| [92] | Bioremediation | - | - | Single | *Achromobacter sp.+*Biochar | PHE | 56.24 | 100 | 43.76 | 30 ± 1 | 3 h | 7 | Yes | Self-spiked |
|  | Nano-Bioremediation | TiO_2_@BC + Xenon lamp | 0.5 g/L | Single | *Achromobacter sp.* +Biochar | PHE | 72.58 | 100 | 27.42 | 30 ± 1 | 3 h | 7 | Yes | Self-spiked |
| [93] | Bioremediation | - | - | Single | *Burkholderia cepacia* | NAP | 67.3 | 20 | 6.54 | 30 | 48 h | 7 | No | Self-spiked |
|  | Nano-Bioremediation | Reduced graphene oxide | 0.3 mg/L | Single | *Burkholderia cepacia* | NAP | 99.0 | 20 | 0.2 | 30 | 48 h | 7 | No | Self-spiked |
| [94] | Bioremediation | - | - | Mix | *Pseudomonas sp. Rhodocucus sp.* (NDH and C2,3D) | ANT | 58.3 | 30 | 12.5 | 30 | 48h | 6.5 | No | Self-spiked |
|  | Nano-Bioremediation | Polyimide aerogels (size nm) | 1g | Mix | *Pseudomonas sp. Rhodocucus sp.* (NDH and C2,3D) | ANT | 78.6 | 30 | 6.4 | 30 | 48h | 6.5 | No | Self-spiked |
| [56] | Bioremediation | - | - | Mix | *Pseudomonas stutzeri* and *Acinetobacter baumannii* | ANT, PYR, BAP | 52.6 | 500 | 237 | 40 | 20d | 7 | No | Self-spiked |
|  | Nano-Bioremediation | Iron nanoparticles | 10 mg/L |  | *Pseudomonas stutzeri* and *Acinetobacter baumannii* | ANT, PYR, BAP | 65.7 | 500 | 171.5 | 40 | 20d | 7 | No | Self-spiked |
|  | Nano-Bioremediation | Iron nanoparticles | 10 mg/L |  | *Pseudomonas stutzeri* and *Acinetobacter baumannii*+ Biosurfactant (*Bacillus subtilis*) | ANT, PYR, BAP | 85 | 500 | 75 | 40 | 20d | 7 | Yes | Self-spiked |
| [95] | Bioremediation | - | - | Single | *Penicillium oxalicum* | PYR | 72 | 20 | 5.6 | 37 | 72 h | NA | Yes | Self-spiked |
|  | Nano-Bioremediation | Carbon nanotube composites | 20 mg in 20ml | Single | *Penicillium oxalicum* | PYR | 90 | 20 | 2 | 37 | 72 h | NA | Yes | Self-spiked |
| [96] | Bioremediation | - | - | Mix | Biofilm from consortia Planococcaceae, Oxalobacteraceae etc | PHE | 12.97 ± 0.44 | 10 | 8.7 | 25 | 24 h | NA | Yes | Self-spiked |
|  | Nano-Bioremediation | Cu, N-TiO_2_ | 0.1 g | Mix | Biofilm from consortia Planococcaceae, Oxalobacteraceae etc | PHE | 88.63 ± 0.71 | 10 | 1.14 | 25 | 24 h | NA | Yes | Self-spiked |
| [97] | Bioremediation | - | - | Mix | Biofilm from consortia Planococcaceae, Oxalobacteraceae etc | PYR | 6.65 ± 0.72 | 1 | 0.335 | 25 | 24 h | NA | Yes | Self-spiked |
|  | Nano-Bioremediation | Cu, N-TiO_2_ | NA | Mix | Biofilm from consortia Planococcaceae, Oxalobacteraceae etc | PYR | 63.89 ± 1.03 | 1 | 0.36 | 25 | 24 h | NA | Yes | Self-spiked |
| [98] | Bioremediation | - | - | Single | *Candida tropicalis* | ICP | 61± 0.04 | 1 | 0.39 | 30 | 15d | 7 | Yes | Self-spiked |
|  | Nano-Bioremediation | Iron nanoparticles | 0.008 g/ L | Single | *Candida tropicalis* | ICP | 75 ± 0.07 | 1 | 0.25 | 30 | 15d | 7 | Yes | Self-spiked |
| [99] | Bioremediation | - | - | Mix | *Rhodotorula sp. Debaryomyces hansenii* and *Hanseniaspora valbyensis* | BghiP | 60.0 | 40 | 16 | 30 | 6d | 7 | No | Self-spiked |
|  | Nano-Bioremediation | ZnO | 0.5 g/L | Mix | *Rhodotorula sp. Debaryomyces hansenii* and *Hanseniaspora valbyensis* | BghiP | 60.7 | 40 | 15.72 | 30 | 6d | 7 | No | Self-spiked |
| [100] | Bioremediation | - | - | Mix | *Rhodotorula sp. Hanseniaspora opuntiae* and *Debaryomyces hansenii* | BAP | 76.0 | 50 | 12 | 30 | 6d | 7 | No | Self-spiked |
|  | Nano-Bioremediation | ZnO | 0.5 g/L | Mix | *Rhodotorula sp. Hanseniaspora opuntiae* and *Debaryomyces hansenii* | BAP | 77.2 | 50 | 11.4 | 30 | 6d | 7 | No | Self-spiked |
| [101] | Bioremediation | - | - | Single | *Bacillus thuringiensis* | PHE | 65.71 | 40 | 13.7 | 34 | 15 d | 7 | No | Self-spiked |
|  | Nano-Bioremediation | MWCNT buckypaper | 7.4 ppm | Single | *Bacillus thuringiensis* | PHE | 93.81 | 40 | 2.47 | 34 | 15 d | 7 | No | Self-spiked |
| [102] | Bioremediation | - | - | Single | *Sphingomonas sp.* | PHE | 74.6 | 100 | 25.4 | 30 | 24 h | 7.2 | No | Self-spiked |
|  | Nano-Bioremediation | Nano bamboo charcoal | 200 mg /L | Single | *Sphingomonas sp.* | PHE | 93.01 | 100 | 6.99 | 30 | 24 h | 7.2 | No | Self-spiked |
| [103] | Bioremediation | - | - | Single | *Paracoccus sp.* | BAP | 60 | 10 | 4 | 30 | 7 d | 7 | No | Self-spiked |
|  | Nano-Bioremediation | Hematite NPs | 5 mg/mL | Single | *Paracoccus sp.* | BAP | 45.8 | 10 | 5.42 | 30 | 7 d | 7 | No | Self-spiked |
| [104] | Bioremediation | - | - | Single | *Sphingomonas sp.* | PHE | 69.83 | 100 | 30.17 | 30 | 18 h | NA | No | Self-spiked |
|  | Nano-Bioremediation | Nano bamboo charcoal | 200 mg/L | Single | *Sphingomonas sp.* | PHE | 94 | 100 | 6 | 30 | 18 h | NA | No | Self-spiked |
| [105] | Bioremediation | - | - | Mix | Methanosarcina and Methanosaeta, Pseudomonas, Cloastridia, and Synergistetes | PYR | 40.8 | 10 | 5.92 | 38±1 | 25 d | 6.59 | Yes | Contaminated+ Self-spiked |
|  | Nano-Bioremediation | FeS | 200 mg/L | Mix | Methanosarcina and Methanosaeta, Pseudomonas, Cloastridia, and Synergistetes | PYR | 77.5 | 10 | 2.26 | 38±1 | 25 d | 6.59 | Yes | Contaminated+ Self-spiked |
|  | Nano-Bioremediation | Magnetic carbon | 200 mg/L | Mix | Methanosarcina and Methanosaeta, Pseudomonas, Cloastridia, and Synergistetes | PYR | 72.1 | 10 | 2.79 | 38±1 | 25 d | 6.59 | Yes | Contaminated+ Self-spiked |
| [106] | Bioremediation | - | - | Mix | Archaea and methanogen | PHE | 60.52 | 25 | 9.60 ± 0.55 | 32 | 49 d | 7.2–7.4 | No | Self-spiked |
|  | Nano-Bioremediation | Magnetite powder (Size in nm) | NA | Mix | Archaea and methanogen | PHE | 70.94 | 25 | 7.46 ± 0.33 | 32 | 49 d | 7.2–7.4 | No | Self-spiked |
|  | Nano-Bioremediation | Nano Fe_3_O_4_ | NA | Mix | Archaea and methanogen | PHE | 70.89 | 25 | 7.37 ± 0.29 | 32 | 49 d | 7.2–7.4 | No | Self-spiked |

d= Day

h= Hour

**Table S2: Detailed Comparative account of Bioremediation and Nano-Bioremediation for PAHs degradation in soil sample**

| References | Remediation method | Type of NPs | Nano Conc. | Single /Mix inoculation | Bioagent | PAHs type | PAHs degradation (%) | Initial Conc. (mg kg^-1^) | Final Conc. (mg kg^-1^) | Temperature (◦C) | Duration (days) | pH | Supplements | Self-spiked PAHs/contaminated |
| --- | --- | --- | --- | --- | --- | --- | --- | --- | --- | --- | --- | --- | --- | --- |
| [43] | Bioremediation | - | - | Single | *Alcaligenes faecalis* | ANT | 24.2 | 500±12 | 379.8± 2.14 | 35 | 30 | 7 | Yes | Self-spiked |
|  | Nano-Bioremediation | TiO_2_ NPs | 150  mg/L | Single | *Alcaligenes faecalis* | ANT | 37.9 | 500±12 | 310± 34.5 | 35 | 30 | 7 | Yes | Self-spiked |
| [107] | Bioremediation (phytoremediation) | - | - | Mix | Proteobacteria, Actinobacteria,Bacteroidota,Firmicutes etc | FLU | 92.60 | 105 ± 18.45 | 7.7 | 15-25 | 150 | 6.7 | Yes | Contaminated+ Self-spiked |
|  | Nano-Bioremediation | Graphene oxide | 0.4 g/Kg | Mix | Proteobacteria, Actinobacteria,Bacteroidota,Firmicutes etc | FLU | 95.57 | 105 ± 18.45 | 4.65 | 15-25 | 150 | 6.7 | Yes | Contaminated+ Self-spiked |
|  | Bioremediation (phytoremediation) | - | - | Mix | Proteobacteria, Actinobacteria,Bacteroidota,Firmicutes etc | PYR | 90.11 | 105 ± 18.45 | 10.38 | 15-25 | 150 | 6.7 | Yes | Contaminated+ Self-spiked |
|  | Nano-Bioremediation | Graphene oxide | 0.4 g/kg | Mix | Proteobacteria, Actinobacteria,Bacteroidota,Firmicutes etc | PYR | 95.88 | 105 ± 18.45 | 4.33 | 15-25 | 150 | 6.7 | Yes | Contaminated+ Self-spiked |
|  | Bioremediation  (phytoremediation) | - | - | Mix | Proteobacteria, Actinobacteria,Bacteroidota,Firmicutes etc | BAA | 87.70 | 105 ± 18.45 | 12.9 | 15-25 | 150 | 6.7 | Yes | Contaminated+ Self-spiked |
|  | Nano-Bioremediation | Graphene oxide | 0.4 g/kg | Mix | Proteobacteria, Actinobacteria,Bacteroidota,Firmicutes etc | BAA | 93.30 | 105 ± 18.45 | 7.03 | 15-25 | 150 | 6.7 | Yes | Contaminated+ Self-spiked |
|  | Bioremediation  (phytoremediation) | - | - | Mix | Proteobacteria, Actinobacteria,Bacteroidota,Firmicutes etc | CHRY | 88.85 | 105 ± 18.45 | 11.7 | 15-25 | 150 | 6.7 | Yes | Contaminated+ Self-spiked |
|  | Nano-Bioremediation | Graphene oxide | 0.4 g/kg | Mix | Proteobacteria, Actinobacteria,Bacteroidota,Firmicutes etc | CHRY | 91.82 | 105 ± 18.45 | 8.59 | 15-25 | 150 | 6.7 | Yes | Contaminated+ Self-spiked |
|  | Bioremediation  (phytoremediation) | - | - | Mix | Proteobacteria, Actinobacteria,Bacteroidota,Firmicutes etc | BBF | 55.50 | 154.55 ± 20.78 | 68.77 | 15-25 | 150 | 6.7 | Yes | Contaminated+ Self-spiked |
|  | Nano-Bioremediation | Graphene oxide | 0.4 g/kg | Mix | Proteobacteria, Actinobacteria,Bacteroidota,Firmicutes etc | BBF | 74.22 | 154.55 ± 20.78 | 29.84 | 15-25 | 150 | 6.7 | Yes | Contaminated+ Self-spiked |
|  | Bioremediation (phytoremediation) | - | - | Mix | Proteobacteria, Actinobacteria,Bacteroidota,Firmicutes etc | BKF | 55.97 | 154.55 ± 20.78 | 68.04 | 15-25 | 150 | 6.7 | Yes | Contaminated+ Self-spiked |
|  | Nano-Bioremediation | Graphene oxide | 0.4 g/kg | Mix | Proteobacteria, Actinobacteria,Bacteroidota,Firmicutes etc | BkF | 62.00 | 154.55 ± 20.78 | 58.72 | 15-25 | 150 | 6.7 | Yes | Contaminated+ Self-spiked |
|  | Bioremediation (phytoremediation) | - | - | Mix | Proteobacteria, Actinobacteria,Bacteroidota,Firmicutes etc | BAP | 56.15 | 154.55 ± 20.78 | 67.77 | 15-25 | 150 | 6.7 | Yes | Contaminated+ Self-spiked |
|  | Nano-Bioremediation | Graphene oxide | 0.4 g/kg | Mix | Proteobacteria, Actinobacteria,Bacteroidota,Firmicutes etc | BAP | 67.37 | 154.55 ± 20.78 | 50.42 | 15-25 | 150 | 6.7 | Yes | Contaminated+ Self-spiked |
|  | Bioremediation (phytoremediation) | - | - | Mix | Proteobacteria, Actinobacteria,Bacteroidota,Firmicutes etc | DBA | 58.03 | 154.55 ± 20.78 | 64.86 | 15-25 | 150 | 6.7 | Yes | Contaminated+ Self-spiked |
|  | Nano-Bioremediation | Graphene oxide | 0.4 g/kg | Mix | Proteobacteria, Actinobacteria,Bacteroidota,Firmicutes etc | DBA | 67.75 | 154.55 ± 20.78 | 49.84 | 15-25 | 150 | 6.7 | Yes | Contaminated+ Self-spiked |
| [108] | Bioremediation | - | - | Mix | Proteobacteria, Acidobacteria, Gemmatimonadota, Bacteroidota (Soil microbes) | PAHs | 19.57 | 299.72+14.07 | 241.06 | NA | 28 | 7.3 | Yes | Contaminated |
|  | Nano-Bioremediation | Graphene oxide | 0–40 mg/kg | Mix | Proteobacteria, Acidobacteria, Gemmatimonadota, Bacteroidota (Soil microbes) | PAHs | 41.07 | 299.72±14.07 | 176.62 | NA | 28 | 7.3 | Yes | Contaminated |
| [109] | Bioremediation | - | - | Mix | *Geobacter* and *Geothrix* | PAHs | 8.7 | 2.479 ± 0.074 | 2.26 ± 0.011 | 20 | 215 | 7 | Yes | Contaminated |
|  | Nano-Bioremediation | Magnetite NPs+biochar | 0.5 g/L | Mix | *Geobacter* and *Geothrix* | PAHs | 86 | 2.479 ± 0.074 | 0.34 ± 0.018 | 20 | 215 | 7 | Yes | Contaminated |
| [110] | Bioremediation | - | - | Single | *Pichia methanolica* (LiP) | PHE | 23.7 | 12 | 9.15 | 25 | 4 | 6 | No | Self-spiked |
|  | Nano-Bioremediation | Chitosan-modified  halloysite nanotubes (HNTs-CTA) | 1g | Single | *Pichia methanolica* (LiP) | PHE | 51.3 | 12 | 5.84 | 25 | 4 | 6 | No | Self-spiked |
|  | Bioremediation | - | - | Single | *Pichia methanolica* (LiP) | FLU | 25 | 12 | 9 | 25 | 4 | 6 | No | Self-spiked |
|  | Nano-Bioremediation | Chitosan-modified  halloysite nanotubes (HNTs-CTA) | 1g | single | *Pichia methanolica* (LiP) | FLU | 38.1 | 12 | 7.43 | 25 | 4 | 6 | No | Self-spiked |
| [111] | Bioremediation | - | - | Mix | Soil Microbes | NAP | 92.7 | 200 | 14.7 | 25 | 30 | 7 | Yes | Contaminated+ Self-spiked |
|  | Nano-Bioremediation | Ag_3_PO_4_@Fe_3_O_4_ (visible light) | 50g/L | Mix | Soil Microbes | NAP | 93.7 | 200 | 12.7 | 25 | 30 | 7 | Yes | Contaminated+ Self-spiked |
|  | Bioremediation | - | - | Mix | Soil Microbes | ANT | 84.8 | 200 | 30.4 | 25 | 30 | 7 | Yes | Contaminated+ Self-spiked |
|  | Nano-Bioremediation | Ag_3_PO_4_@Fe_3_O_4_ | 50g/L | Mix | Soil Microbes | ANT | 91.9 | 200 | 16.2 | 25 | 30 | 7 | Yes | Contaminated+ Self-spiked |
|  | Bioremediation | - | - | Mix | Soil Microbes | PHE | 86 | 200 | 28.0 | 25 | 30 | 7 | Yes | Contaminated+ Self-spiked |
|  | Nano-Bioremediation | Ag_3_PO_4_@Fe_3_O_4_ | 50g/L | Mix | Soil Microbes | PHE | 94.6 | 200 | 10.8 | 25 | 30 | 7 | Yes | Contaminated+ Self-spiked |
|  | Bioremediation | - | - | Mix | Soil Microbes | FLU | 68.6 | 200 | 62.8 | 25 | 30 | 7 | Yes | Contaminated+ Self-spiked |
|  | Nano-Bioremediation | Ag_3_PO_4_@Fe_3_O_4_ | 50g/L | Mix | Soil Microbes | FLU | 82.3 | 200 | 35.5 | 25 | 30 | 7 | Yes | Contaminated+ Self-spiked |
|  | Bioremediation | - | - | Mix | Soil Microbes | PYR | 63.4 | 200 | 73.3 | 25 | 30 | 7 | Yes | Contaminated+ Self-spiked |
|  | Nano-Bioremediation | Ag_3_PO_4_@Fe_3_O_4_ | 50g/L | Mix | Soil Microbes | PYR | 78.8 | 200 | 42.4 | 25 | 30 | 7 | Yes | Contaminated+ Self-spiked |
| [65] | Bioremediation | - | - | Single | *Paracoccus aminovorans* + Soil microbes | PAHs | 46.9 | 210.01 | 111.51 | 30 | 35 | 8.32 | Yes | Contaminated |
|  | Nano-Bioremediation | Graphene oxide | 0.1 g/ml | Single | *Paracoccus aminovorans*  + Soil microbes | PAHs | 62.86 | 210.01 | 77.99 | 30 | 35 | 8.32 | Yes | Contaminated |
| [112] | Bioremediation | - | - | Mix | *Bacillus cereus, Acidovorax wohlfahrtii,* and *Bacillus thuringiensis* | PYR | 88 | 100 | 12±3.4 | 30 | 35 | 7 | Yes | Contaminated+ Self-spiked |
|  | Nano-Bioremediation | Hematite NPs | 4.7 g/L | Mix | *Bacillus cereus, Acidovorax wohlfahrtii,* and *Bacillus thuringiensis* | PYR | 96 | 100 | 4 ±1.5 | 30 | 35 | 7 | Yes | Contaminated+ Self-spiked |

d= Day

h= Hour
